# Exploring Vitamin D Signaling‐Associated Biomarkers and Their Diagnostic Value in Diabetic Retinopathy: A Combined Transcriptomic and Single‐Cell Analysis With Experimental Validation

**DOI:** 10.1155/jdr/7240252

**Published:** 2026-04-30

**Authors:** Pengfei Chen, Ruiqi Li, Keren Zhao, Rui Liu, Yuhua Hao

**Affiliations:** ^1^ Department of Ophthalmology, The Fourth Hospital of Hebei Medical University, Shijiazhuang, 050000, China, hebmu.edu.cn

**Keywords:** diabetic retinopathy, single-cell RNA sequencing, transcriptomics, vitamin D signaling-associated downstream biomarkers

## Abstract

**Background:**

Diabetic retinopathy (DR) can significantly impair vision and lead to blindness. Vitamin D (VD) has been shown to enhance the production of anti‐inflammatory factors, alleviating the effects of hyperglycemia. However, downstream genes and molecular networks associated with VD signaling in DR remain unidentified. This study aimed to employ a systems biology approach to nominate high‐priority candidate genes and cellular contexts as a hypothesis‐generating effort to facilitate future functional studies on the role of VD in DR.

**Methods:**

DR‐related datasets were obtained from public databases to identify differentially expressed genes (DEGs). Seven canonical VD metabolism‐related genes (VDRGs) were subjected to weighted gene co‐expression network analysis (WGCNA) to identify VD signaling‐associated model genes. Candidate genes were selected based on the intersection of DEGs and model genes. “Boruta” and support vector machine–recursive feature elimination (SVM–RFE), along with expression validation, were used to screen for biomarkers. Further analyses included immune infiltration, gene set enrichment analysis (GSEA), regulatory network construction, and drug prediction. Single‐cell RNA sequencing (scRNA‐seq) was utilized to assess cellular heterogeneity, identifying distinct cell clusters and key cells based on gene expression profiles. Cell–cell communication within immune cells was also examined. Biomarker expression levels in clinical samples were validated through real‐time reverse transcription polymerase chain reaction (RT‐qPCR).

**Results:**

The biomarkers SLC36A1 and RAB23 were identified as VD signaling‐associated downstream candidates and validated. GSEA revealed their primary association with glucose metabolism. B cells and CD4 T cells were identified as differentially expressed immune cells. Both biomarkers were regulated by a competing endogenous RNA (ceRNA) network, and the drug “methyl methanesulfonate” targeted both biomarkers simultaneously. Single‐cell analysis identified 11 distinct cell types, including classical monocytes, B cells, and T cells. B cells and classical monocytes were identified as key cells due to the differential expression of biomarkers. The cell–cell communication network highlighted interactions, particularly between classical monocytes, B cells, and T cells. The differentiation of key cells and the stage of biomarker expression were also uncovered. RT‐qPCR analysis revealed a significant upregulation of SLC36A1 and RAB23 in the DR group compared to controls (*F* = 5.184 *p* = 0.027 < 0.05; *F* = 4.147 *p* = 0.047 < 0.05).

**Conclusion:**

SLC36A1 and RAB23 were identified as VD signaling‐associated downstream biomarkers in DR, providing a framework for exploring the potential link between VD signaling and DR pathogenesis through these candidate genes.

## 1. Introduction

Diabetic retinopathy (DR) is one of the most prevalent chronic complications of diabetes mellitus [1], characterized by a series of pathological changes resulting from retinal microvascular damage due to hyperglycemia. It is a progressive condition that can impair vision and even lead to blindness. The global prevalence of DR among diabetic patients is 22.27% [2]. Hyperglycemia contributes to the development of DR by increasing retinal vascular permeability, leading to fluid accumulation in and beneath the retina, which causes neuronal edema and necrosis [3]. Furthermore, chronic retinal inflammation induced by hyperglycemia has been implicated in the progression of DR [4]. The pathophysiology of DR is intricate, and its underlying mechanisms remain incompletely understood. In diabetic individuals, elevated intracellular glucose levels activate the polyol pathway, which metabolizes glucose and results in the accumulation of advanced glycation end‐products (AGEs), protein kinase C (PKC) activation, and upregulation of the AGE receptor and hexokinase pathways [5, 6]. Additionally, levels of chemokines (e.g., MCP‐1, CCL2, and CCL5) and pro‐inflammatory cytokines (such as TNF‐α, IL‐1β, and IL‐6) are significantly elevated in DR compared to normal samples [7]. Early intervention is critical to prevent or delay the progression of DR. Therefore, ongoing research into the pathogenesis of DR and the identification of effective therapeutic targets are crucial.

Vitamin D (VD) is a fat‐soluble steroid, primarily composed of VD2 and VD3 [8]. Its main role is to maintain normal calcium levels and support bone development by regulating phosphate and calcium metabolism [9]. VD has also been shown to reduce the risk of cardiometabolic diseases, including hypertension, cardiovascular disease, and diabetes [8]. Studies have indicated a link between VD deficiency and the severity of DR [10]. VD may mitigate DR or reduce its incidence by decreasing oxidative stress, modulating inflammation and immune responses, lowering the production of transforming growth factor‐beta (TGF‐β, a biomarker of DR), inhibiting the renin–angiotensin–aldosterone system (RAAS), alleviating endoplasmic reticulum stress, and regulating endothelial cell apoptosis [11]. Additionally, VD may reduce hyperglycemia‐induced retinal vascular damage and cell apoptosis by decreasing the expression of reactive oxygen species and vascular endothelial growth factor (VEGF) in retinal cells [12]. However, the mechanisms of VD metabolism‐related genes (VDRGs) in DR, as well as their diagnostic potential, remain unexplored. Thus, further investigation into the relationship between VD metabolism and DR is necessary.

Single‐cell RNA sequencing (scRNA‐seq) enables the analysis of tissue distribution and functional states of different cell types at the single‐cell level [13]. This approach significantly enhances the resolution and accuracy of data, allowing for the identification of cellular heterogeneity, the observation of dynamic changes in cell states during disease progression, and the exploration of individual variations in therapeutic responses [14].

This study integrated transcriptomic data from public databases with existing literature and utilized weighted gene co‐expression network analysis (WGCNA) and machine learning to identify candidate VDRGs in DR. The expression levels of candidate genes were analyzed using the Wilcoxon rank‐sum test (*p*  < 0.05), leading to the identification of key genes. Comprehensive analyses of the signaling pathways, subcellular localization, protein structure predictions, and immune infiltration levels of these biomarkers were conducted. Finally, single‐cell analysis was applied to map the cell type‐specific expression and distribution patterns of these biomarker genes in DR. This study adopts a systems biology approach with a hypothesis‐generating purpose: to nominate high‐priority candidate genes and their cellular contexts, rather than to establish definitive mechanistic conclusions. The findings are intended to provide focused, testable targets for future functional research on the role of VD in DR.

## 2. Materials and Methods

### 2.1. Data Extraction

Two DR datasets, namely GSE221521 (GPL24676 platform) and GSE189005 (GPL23126 platform), were retrieved from the Gene Expression Omnibus (GEO) database (https://www.ncbi.nlm.nih.gov/geo/database). The GSE221521 dataset, which includes 50 control and 69 DR samples, was used as the training set, while GSE189005 (comprising 9 control and 10 DR samples) served as the validation set. Seven VDRGs (DHCR7, VDBP, CYP2R1, CYP27A1, CYP24A1, CYP27B1, and VDR) were extracted from two published studies [15, 16]. Furthermore, single‐cell transcriptomic data (GSE248284) from the GEO database, containing three DR and three control (NDR) samples from peripheral blood mononuclear cells (PBMCs) (GPL24676 platform), were also utilized.

### 2.2. Screening of Candidate Genes

The “DESeq2” package (v 1.38.0) [17] was used to identify differentially expressed genes (DEGs) between DR and control groups in the GSE221521 training set, applying criteria of |log_2_ fold change (FC)| > 1 and *p*  < 0.05. The “ggplot2” package (v 3.4.1) [18] and “ComplexHeatmap” package (v 2.14.0) [19] were employed to visualize the DEGs through volcano plots and heatmaps, respectively. Next, single‐sample gene set enrichment analysis (ssGSEA) was conducted for the 7 VDRGs using the “GSVA” package (v 1.46.0) [20], scoring each VDRG. Wilcoxon tests (*p*  < 0.05) were used to compare the ssGSEA scores of VDRGs between DR and control groups. To identify model genes with the highest correlation to ssGSEA scores, WGCNA was performed on the training set. In the WGCNA process, unqualified samples were removed (cutHeight = 160), and sample clustering was performed using the “goodSamplesGenes” and “hclust” functions in the “WGCNA” package (v 1.71) [21]. To construct a scale‐free gene co‐expression network, the “pickSoftThreshold” function was used to analyze parameters of different soft‐threshold powers (β). Finally, the optimal β was selected based on the criteria that the scale‐free topology fitting index (*R* ^2^) of the network reached 0.8 while maintaining a high mean gene connectivity, indicating that the network not only conforms to the characteristics of an approximate scale‐free distribution but also avoids information loss caused by excessively low connectivity. Genes were then grouped into different modules, with a minimum of 100 genes per module and a module fusion threshold (mergeCutHeight) of 0.4. “Pearson” correlations between module eigengene (ME) scores and ssGSEA scores were computed (|cor| > 0.4, *p*  < 0.05), and modules with the highest correlation were selected as candidate model genes. The final candidate genes were identified by intersecting the module genes with DEGs using the “ggvenn” package (v 0.1.9) [22].

### 2.3. Enrichment Analyses of Candidate Genes and Protein–Protein Interaction (PPI)

To explore the biological functions and pathways of the candidate genes, Gene Ontology (GO) and Kyoto Encyclopedia of Genes and Genomes (KEGG) enrichment analyses were conducted using the “clusterProfiler” package (v 4.2.2) [23], with a significance threshold of *p*  < 0.05. GO enrichment analysis was categorized into biological processes (BPs), cellular components (CCs), and molecular functions (MFs), and the top 10 GO terms were presented. Additionally, to investigate the protein‐level interactions of the candidate genes, the genes were uploaded to the STRING database (http://string-db.org/) for PPI network construction, using a confidence threshold of 0.400. The resulting network was visualized using “Cytoscape” (v 3.7.2) [24].

### 2.4. Identification of Biomarkers

To identify candidate biomarkers from candidate genes, two machine learning methods were utilized. Boruta, a feature selection technique based on the random forest algorithm, was employed to mitigate the effects of random fluctuations and correlations. This analysis was conducted using the “Boruta” package (v 8.0.0) [25]. Additionally, support vector machine–recursive feature elimination (SVM–RFE), performed via the “caret” package (v 6.0‐93) [26], was used to assess the importance of each gene by ranking them based on error rates and accuracy at each iteration. Genes were selected when the accuracy reached its peak. The genes selected by Boruta and SVM–RFE were intersected to identify potential biomarkers. The expression levels of these candidate biomarkers were then validated in both the training and validation sets. Biomarkers that exhibited differential expression with consistent trends across the datasets were considered as valid biomarkers. To evaluate the diagnostic potential of the biomarkers, receiver operating characteristic (ROC) curve analysis was performed using the pROC package (v1.18.0) [27] on the combined GSE221521 and GSE189005 datasets. The area under the curve (AUC) was calculated for each gene, with values greater than 0.5 indicating some diagnostic capacity, and those exceeding 0.7 representing good to excellent diagnostic ability. This analysis also included the validation of biomarker expression levels in the GSE189005 dataset.

### 2.5. Gene Set Enrichment Analysis (GSEA) of Biomarkers

Next, GSEA was conducted to identify biological pathways associated with the biomarkers. Pearson correlation coefficients between each biomarker and all genes in the training set were computed, and genes were ranked according to these coefficients. GSEA was then performed using the “clusterProfiler” package (v 4.2.2), with thresholds set at adj. *p*  < 0.05 and |normalized enrichment score (NES)| > 1. The “c2.cp.kegg.v2023.1.Hs.symbols.gmt” gene set was used as the reference, and the top 5 results were plotted using the “enrichplot” package (v 1.18.3) [28].

### 2.6. Protein Structure Prediction, Subcellular Localization, and Disease Correlation Analyses

To further investigate the protein structures and amino acid sequences of the biomarkers, their human protein sequence numbers and amino acid sequences were retrieved from the Uniprot database (https://www.uniprot.org/). The sequence data were then used to predict protein structures through the AlphaFold2 website (https://alphafold.ebi.ac.uk/search). Additionally, the amino acid sequences were uploaded to the Hum‐mPLoc 3.0 server (http://www.csbio.sjtu.edu.cn/) to determine their subcellular localization. Finally, the CTD database (https://ctdbase.org/) was used to explore potential links between the biomarkers and other diseases.

### 2.7. Analysis of Immune Infiltration

To explore the immune system’s role in DR, differential immune cell infiltration levels between the DR and control groups were assessed using the “epic” algorithm, included in the “IOBR” package (v 0.99.9) [29]. Wilcoxon tests were applied to compare immune cell infiltration levels between the two groups (*p*  < 0.05), and differential immune cells were identified. Spearman correlation analysis was then performed to determine whether biomarkers were associated with the differential immune cells (*p*  < 0.05).

### 2.8. Establishment of Gene–Gene Interaction (GGI) Network, Two Regulatory Networks Along With Drug Prediction Network

To further investigate the biomarkers, four networks were established. First, a GGI network was constructed using the GeneMANIA website (http://genemania.org/) to explore the relationships between biomarkers and other genes. Second, to analyze potential competing endogenous RNAs (ceRNAs), miRNAs predicted to interact with biomarkers were obtained from the miRWalk (http://mirwalk.umm.uni-heidelberg.de/search_genes/) and miRTarBase (https://mirtarbase.cuhk.edu.cn/~miRTarBase/miRTarBase_2022/php/search.php) databases. The miRNAs from these sources were then intersected to identify relevant miRNAs. Based on these miRNAs, long noncoding RNAs (lncRNAs) were searched in the ENCORI (https://starbase.sysu.edu.cn/agoClipRNA.php?source=mRNA) database (CLIP‐Data ≥ 1, Degradome‐Data ≥ 0, pan‐Cancer ≥ 0) and the miRNet (https://www.mirnet.ca/) database, and the results were intersected to construct a ceRNA (lncRNA–miRNA–mRNA) regulatory network. Third, potential transcription factors (TFs) for biomarkers were identified using the NetworkAnalyst website (https://www.networkanalyst.ca/NetworkAnalyst/uploads/ListUploadView.xhtml) to build a TF–mRNA network, with the organism set as “*H. sapiens* (human)” and using the ENCODE database. Finally, to identify potential drugs targeting the biomarkers, the “enrichR” package (v 3.1) [30] in combination with the drug signature database (DsigDB) (https://dsigdb.tanlab.org/DSigDBv1.0) was used (*p*  < 0.05), and the prediction results were visualized using “Cytoscape” (v 3.7.2).

### 2.9. Single‐Cell Data Process

For single‐cell analysis, the “CreateSeuratObject” function in the “Seurat” package (v 5.0.1) [31] was used to convert the “GSE248284” single‐cell transcriptome data into a Seurat object for quality control (QC). The detected genes per cell (nFeature_RNA) and total RNA counts per cell (nCount_RNA) were visualized using the “ggplot2” package (v 3.4.3). Cells with nFeature_RNA ≤200 or ≥5000, genes with nCount_RNA ≤200 or ≥30,000, as well as genes covered by fewer than 3 cells and cells containing fewer than 200 genes, were excluded. Notably, the GSE248284 dataset lacks detailed clinical metadata for sample stratification. Preliminary analysis indicated the presence of sample‐level clustering, which may reflect unrecorded sample heterogeneity such as technical batch effects or unknown clinical factors. To ensure the robustness of the analytical results, our subsequent analyses focused on conserved cell types consistently identified across all samples, thereby minimizing the impact of uncontrolled heterogeneity on cell‐type‐specific conclusions. The “NormalizeData” and “FindVariableFeatures” functions were used to harmonize, normalize, and standardize the data. The top 2000 most variable genes identified by the “FindVariableFeatures” function were visualized with “LabelPoints” and exported for further analysis. To standardize the data for subsequent analyses, the “ScaleData” function was applied. Principal component analysis (PCA) was performed to cluster the highly variable genes using the “Jackstraw” function (*p*  < 0.05), with the results visualized using “JackStrawPlot” and “ElbowPlot” functions. The components obtained from PCA were then used for unsupervised clustering with the “FindNeighbors” and “FindClusters” functions in the “Seurat” package (v 5.0.1), followed by uniform manifold approximation and projection (UMAP) clustering using the “UMAP” function (resolution = 0.4), and the cell clusters were visualized with the “DimPlot” function.

### 2.10. Cell Annotation and Identification of Key Cell Clusters

First, marker genes for cell clusters were identified using the “FindAllMarkers” function in the “Seurat” package (v 5.0.1). The Wilcoxon test was applied to detect differential marker genes in cell clusters, with the criteria of |log_2_FC| > 1 and adj.*p*  < 0.05. The top 3 differential markers were visualized using the “ggplot2” package (v 3.4.3). Published marker genes from a prior study [32] were used to identify and annotate cell types within the clusters, excluding markers that appeared in multiple cells. The expression of marker genes in the cell clusters was then visualized using the “VlnPlot” function, and the proportions of cell types in DR and all samples were represented using the “ggpie” package (v 0.2.5) (https://github.com/showteeth/ggpie) and “ggplot2” (v 3.4.3).

Next, key cells were screened by identifying marker genes for each cell type using the “FindAllMarkers” function. Differential markers were selected *via* the Wilcoxon test (|log_2_FC| > 1.2, adj. *p*  < 0.05) and subjected to GO enrichment analysis using the “clusterProfiler” package (v 4.2.2). The top 20 expressed markers for each cell type were presented. The distribution and expression levels of biomarkers in DR samples from the GSE248284 dataset were then visualized using “UMAP” and “VlnPlot” functions, with cell clusters showing the highest biomarker expression identified as key cells.

### 2.11. Cell–Cell Communication and Pseudotime Analysis of Key Cells

Subsequently, the “CellChat” package (v 1.6.1) [33] was used to analyze cell–cell communication at the molecular level in the GSE248284 dataset, using “CellChatDB.human” as the reference. The correlation between different cell types and ligand–receptor interactions were also explored, with results visualized using “ggplot2” (v 3.4.3). To understand the differentiation process of key cells and the expression of biomarkers at different stages of differentiation, key cell clusters were further clustered into subgroups using dimensionality reduction techniques like PCA and UMAP ("Jackstraw” and “UMAP” functions). Finally, pseudotime analysis was conducted using the “Monocle2” package (v 2.24.0) [34], and the results were visualized with the “plot pseudo‐time heatmap” function.

### 2.12. Experimental Validation

The expression of biomarkers in clinical samples was validated using real‐time reverse transcription polymerase chain reaction (RT‐qPCR). A total of 48 blood samples were collected from both the DR group and the normal control group at our hospital between September 1, 2024 and December 1, 2025. It was approved by Research Ethics Committee of the Fourth Hospital of Hebei Medical University (2024KS100), and was conducted in accordance with the tenets of the Declaration of Helsinki. Informed consent was obtained from all participants.

Inclusion criteria were as follows: (1) diagnosis of DR by fundus fluorescein angiography (FFA); (2) no history of hypertension, cardiovascular disease, or other serious systemic conditions. Exclusion criteria were as follows: (1) refractive error < −6 D, axial length (AL) > 26.0 mm; (2) intraocular pressure > 21 mmHg (1 mmHg = 0.133 kPa); (3) history of ocular trauma; (4) presence of other ocular diseases, including glaucoma, uveitis, retinal vascular occlusion, epiretinal membrane, or inflammatory retinal and choroidal conditions.

PBMCs were isolated from the blood samples, and total RNA was extracted using Triquick reagent. The RNA was then reverse‐transcribed into cDNA with the a‐capacity cDNA RT kit, supplemented with RNase inhibitor. Gene‐specific primers (Table 1) were used for subsequent reactions. The reaction mixture, with a total volume of 10 µL, included 1 µL of cDNA, 0.2 µL of each 10 µM forward and reverse primer, 3.6 µL of nuclease‐free water, and 5 µL of reaction mix. The PCR conditions were as follows: initial denaturation at 95°C for 2 min, followed by 40 cycles of denaturation at 95°C for 30 s, annealing at 60°C for 30 s, and extension at 72°C for 30 s. RT‐qPCR was performed in triplicate for each gene to reduce variability and obtain average values at each time point. Threshold cycle (Ct) efficiency was corrected, and melting curves were generated for each gene’s PCR detection. Results were quantified using the comparative Ct method (2^−∆∆Ct^ method, where ∆∆Ct = ∆Ct [sample] − ∆Ct [reference gene]).

**Table 1:** List of primers used for RT‐PCR analysis.

Primer	The sequence (5^’^–3^’^)
Human β‐actin‐F	CACCATTGGCAATGAGCGGTTC
Human β‐actin‐R	AGGTCTTTGCGGATGTCCACGT
SLC36A1‐F	GTCACCATCCTCTACATCAGCC
SLC36A1‐R	CGTAGAACTGGAGTGCGTAGGT
RAB23‐F	GTAGCCGAAGTGGGAGATATACC
RAB23‐R	ACCTTTTTGCCAGTGCCTCAGC

### 2.13. Statistical Analysis

All bioinformatics analyses were performed using “R” (v 4.2.2) software, with a significance level set at *p*  < 0.05.

## 3. Results

### 3.1. 239 Candidate Genes Identified

A total of 1020 DEGs, including 654 upregulated (e.g., CYP26B1, CDH7, and FOXP2) and 366 downregulated (e.g., CTAG2, TP53TG3B, and KRTAP4−6) genes, were identified from 119 samples in the training set (Figure 1A). The distribution of the top 20 DEGs is shown in Figure 1B. The ssGSEA scores of VDRGs between the DR and control groups were significantly different (Figure 1C). Before performing WGCNA, four outlier samples were excluded (Figure S1A), and the remaining 115 samples were clustered (Figure S1B). The optimal threshold “β” was set at 17 based on the scale‐free fit index (*R* ^2^ = 0.806) and mean connectivity (Figure 1D). A gene co‐expression network was constructed, and genes were clustered into 11 modules (Figure 1E). Furthermore, the ME scores of two modules (brown and turquoise) showed a high correlation with the ssGSEA scores (|cor| > 0.4, *p*  < 0.05), indicating that 6759 genes in these modules might be associated with VDRGs (Figure 1F). These 6759 genes were recognized as WGCNA model genes and were used for subsequent analyses. After identifying DEGs and WGCNA model genes, they were intersected, resulting in 239 candidate genes (Figure 1G).

Figure 1(A) Volcano plot of DEGs between the DR group and control group in the training set (GSE221521). Blue dots represent downregulated genes, yellow dots represent upregulated genes (−log_10_ (0.05) = 1.3, *p*  < 0.05). (B) Heatmap of the top 20 DEGs showing distinct expression patterns between DR and control samples in the training set (GSE221521). (C) Violin plot of ssGSEA scores for VDRGs in DR (orange) and control (blue) groups in the training set (GSE221521). (D) Selection of the soft threshold (power) for WGCNA: left panel shows scale independence (*R* ^2^ = 0.806 at power = 17), right panel shows mean connectivity. (E) Cluster dendrogram of genes in the training set (GSE221521), with color bars indicating the 11 co‐expression modules identified by WGCNA. (F) Heatmap of Pearson correlations between MEs and VDRG ssGSEA scores in the training set (GSE221521). Modules with |cor| > 0.4 and *p*  < 0.05 were selected as model genes. (G) Venn diagram showing the intersection of DEGs (from training set GSE221521) and WGCNA model genes (brown and turquoise modules), yielding 239 candidate genes.  ^∗^ *p* < 0.05.

**Figure:**
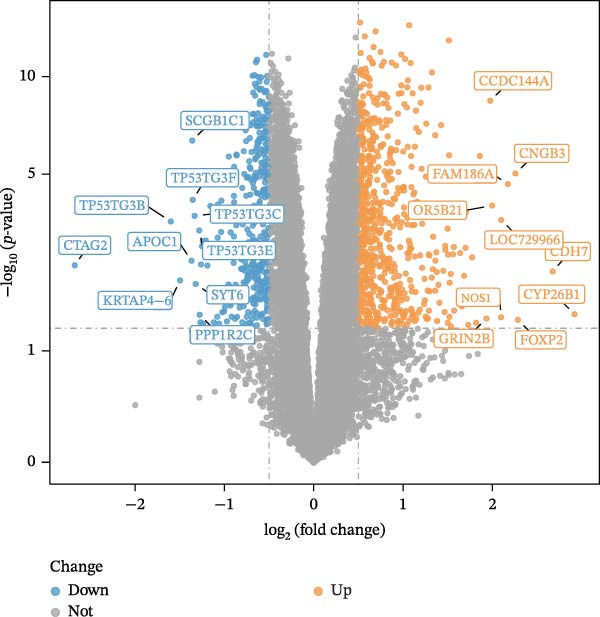
(A)

**Figure:**
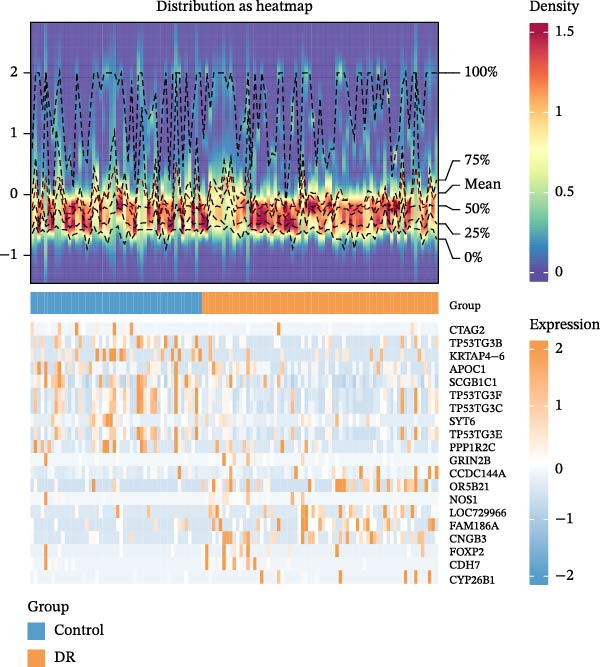
(B)

**Figure:**
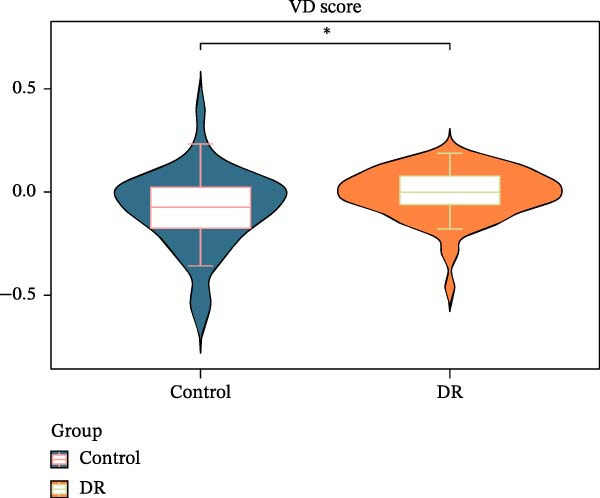
(C)

**Figure:**
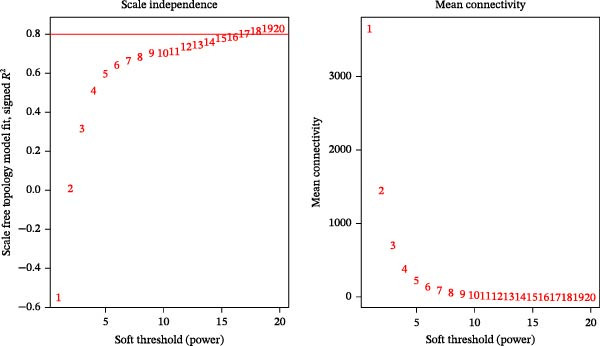
(D)

**Figure:**
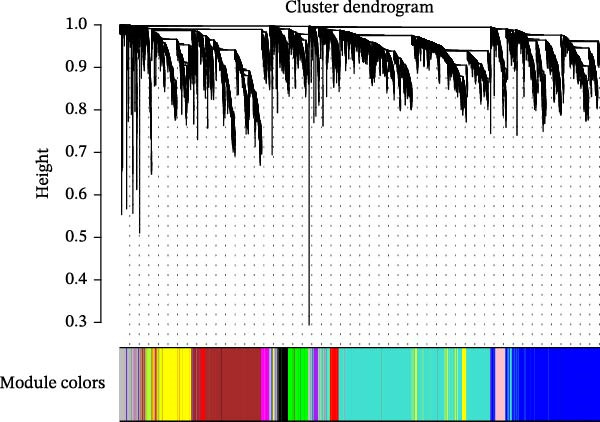
(E)

**Figure:**
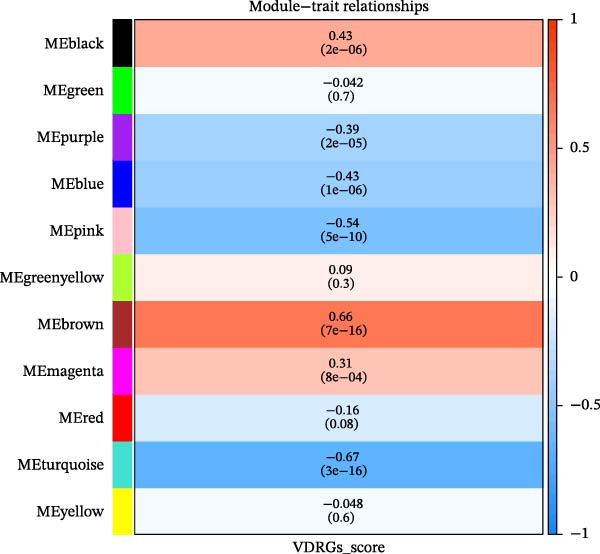
(F)

**Figure:**
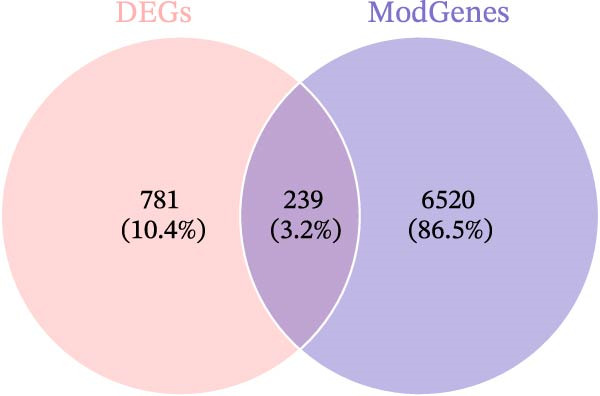
(G)

### 3.2. Candidate Genes Yield 368 GO Entries, 7 KEGG Pathways, and a PPI Network

Functional analysis of the candidate genes revealed enrichment in 368 GO terms, including 315 BPs, 23 CCs, and 30 MFs. The most significantly enriched terms included pro‐B cell differentiation, regulation of lymphoid progenitor cell differentiation, and monocyte activation (Figure 2A), suggesting the involvement of immune cells in VD metabolism in DR. Additionally, seven KEGG pathways were enriched, including ribosome, steroid hormone biosynthesis, and notch signaling pathway (Figure 2B). The PPI network demonstrated that 112 candidate genes had interactions (Figure 2C). Among them, IL10 and CTLA4 exhibited the most complex interactions, and genes without protein interactions were excluded.

Figure 2(A) GO enrichment chord diagram showing the top 10 BPs, CCs, and MFs enriched in candidate genes. The chord length represents the number of genes associated with each term. (B) KEGG enrichment chord diagram of gene‐pathway interactions for candidate genes, highlighting the top 7 significantly enriched pathways (*p*  < 0.05). (C) PPI network of candidate genes (confidence threshold = 0.400). Nodes represent proteins, and edges represent protein–protein interactions.

**Figure:**
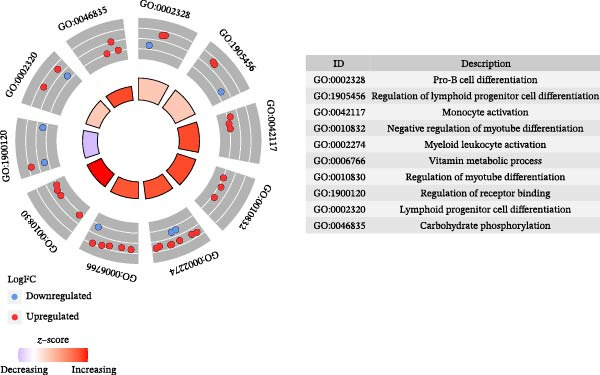
(A)

**Figure:**
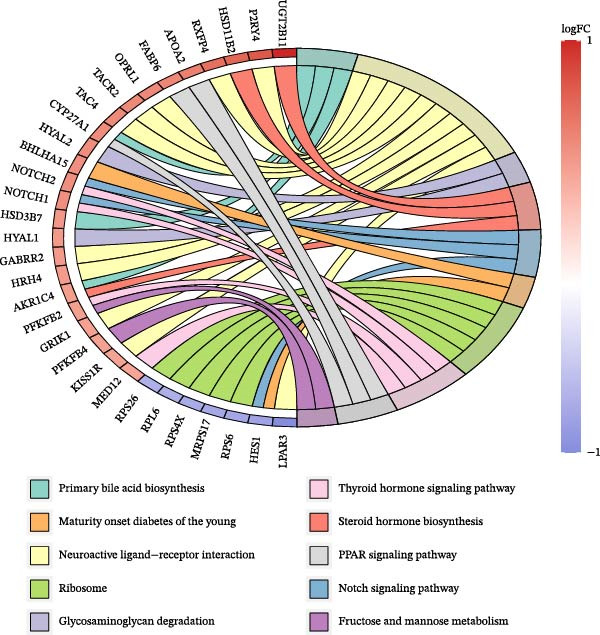
(B)

**Figure:**
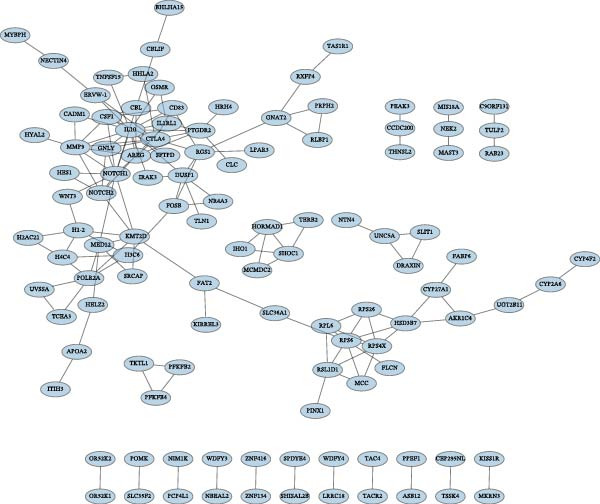
(C)

### 3.3. RAB23 and SLC36A1 Defined as VD Signaling‐Associated Downstream Biomarkers and Biomarkers’ GSEA Results

Two machine learning methods were then applied to select biomarkers from the 112 candidate genes. The SVM–RFE algorithm identified 75 genes when the accuracy reached 0.815942 (Figure 3A). Additionally, 20 genes were shortlisted using the Boruta algorithm (Figure 3B,C). The genes identified by both methods were intersected to obtain 20 candidate biomarkers (Figure 3D). However, only RAB23 and SLC36A1 exhibited significantly different expression levels between DR and control samples in both the training set (GSE221521) and the validation set (GSE189005), with consistent expression trends across both datasets (Figure 3E). Consequently, RAB23 and SLC36A1 were selected as biomarkers. ROC curve analysis revealed distinct diagnostic performances for the two biomarkers. SLC36A1 demonstrated strong diagnostic performance in both the training cohort (GSE221521, AUC = 0.781) and the validation cohort (GSE189005, AUC = 0.956). In contrast, RAB23 exhibited moderate diagnostic power in GSE221521 (AUC = 0.671) but outstanding diagnostic value in GSE189005 (AUC = 0.978) (Figure S2). Subsequently, GSEA was performed on the two biomarkers. The results suggested that RAB23 was mainly involved in glycosylphosphatidylinositol (GPI) anchor biosynthesis, inositol phosphate metabolism, and glycan biosynthesis (Figure 3F). SLC36A1 was primarily associated with organelles such as the proteasome and ribosome, along with oxidative phosphorylation (Figure 3G). GSEA results indicated that both biomarkers may be related to glucose metabolism.

Figure 3(A) Accuracy curve of support vector machine–recursive feature elimination (SVM–RFE) algorithm. (B) Feature importance plot of the Boruta algorithm, showing the top 20 genes with the highest importance scores. (C) Importance ranking of candidate genes by the Boruta algorithm, with blue dots representing confirmed important genes and red dots representing rejected genes. (D) Venn diagram of overlapping genes identified by SVM–RFE and Boruta, yielding 20 candidate biomarkers. (E) Boxplots of biomarker expression in the training set (GSE221521, left) and validation set (GSE189005, right). Orange represents DR samples, while blue represents control samples. (F, G) GSEA of RAB23 and SLC36A1 in the training set (GSE221521). The top 5 enriched pathways are shown.  ^∗∗∗^ *p* < 0.001,  ^∗∗∗∗^ *p* < 0.0001.

**Figure:**
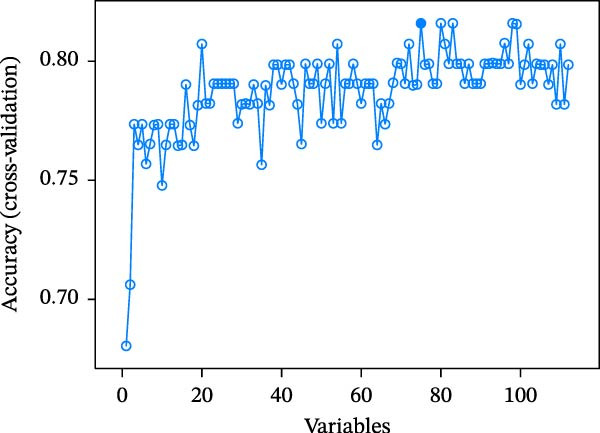
(A)

**Figure:**
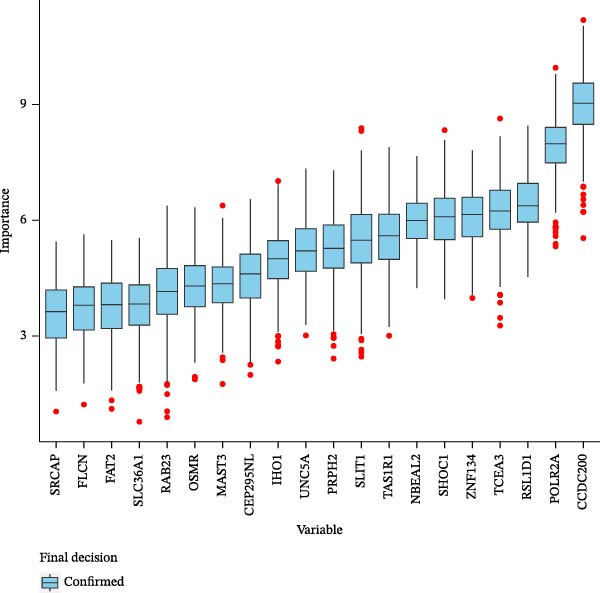
(B)

**Figure:**
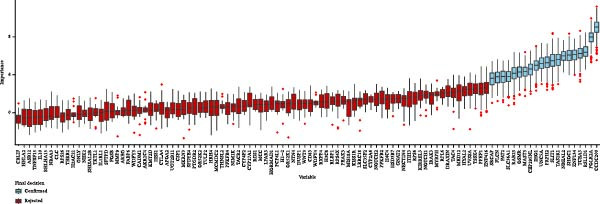
(C)

**Figure:**
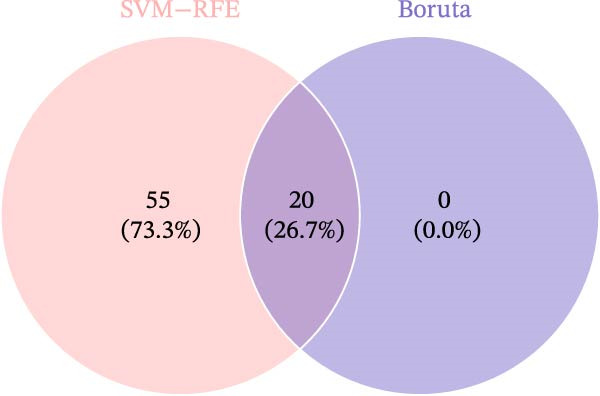
(D)

**Figure:**
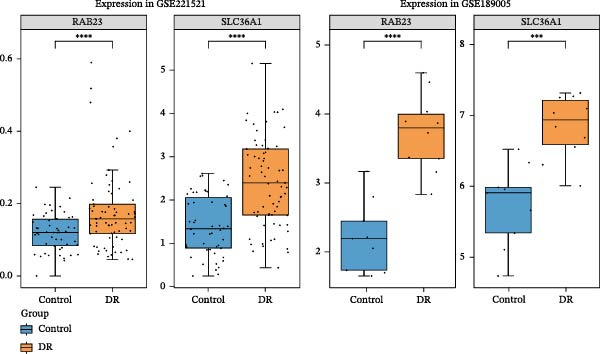
(E)

**Figure:**
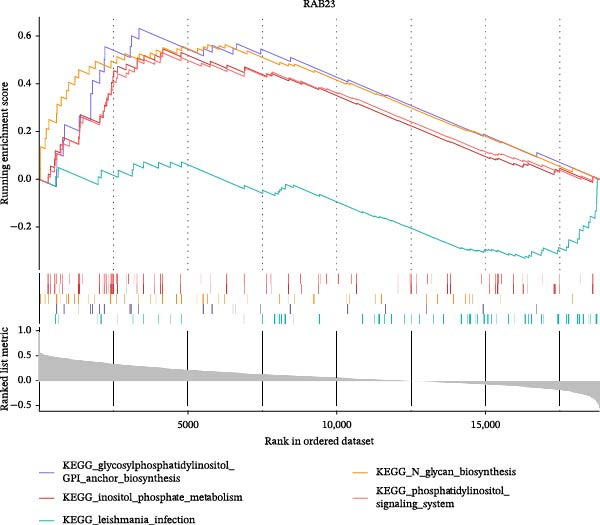
(F)

**Figure:**
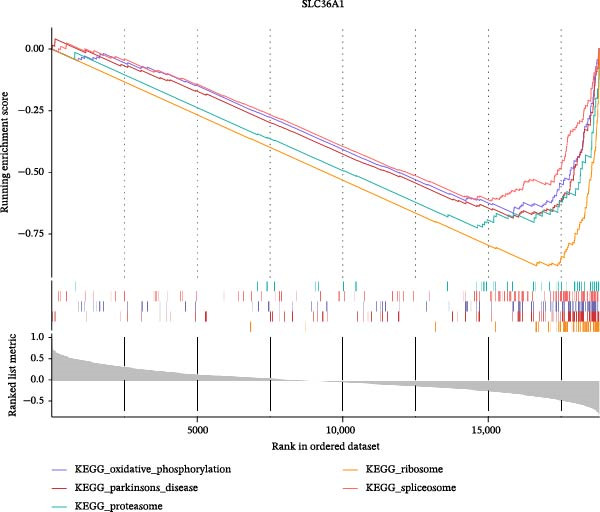
(G)

### 3.4. Protein Structure, Subcellular Localization, and Disease Correlation of RAB23 and SLC36A1

After protein structure prediction, the spatial structure and amino acid sequences of RAB23 (Figure S3A,B) and SLC36A1 (Figure S3C,D) were determined. SLC36A1 was localized in the lysosome and plasma membrane (Figure S3E), while RAB23 was localized in the cytoplasm (Figure S3F). Disease correlation analysis indicated that both RAB23 and SLC36A1 were associated with chemical and drug‐induced liver injury, necrosis, prenatal exposure delayed effects, and weight loss. Additionally, RAB23 was linked to hepatomegaly, while SLC36A1 was associated with inflammation (Figure S3G).

### 3.5. The Association Between Three Differential Immune Cells and Two Biomarkers

In immune infiltration analysis, the infiltration levels of eight types of immune cells are shown in Figure 4A. B cells, CD4 T cells, and an unclassified cell type labeled “other cells” were identified as differential immune cells between the DR and control groups (Figure 4B). Correlation analysis revealed that SLC36A1 was negatively correlated with B cells and positively correlated with “other cells,” while RAB23 was negatively correlated with “other cells” and positively correlated with CD4 T cells (Figure 4C). The relationships between the two biomarkers and the three immune cell types are shown in Figure S4A–F.

Figure 4(A) Stacked bar plot showing the relative abundance of immune cell subpopulations in individual DR and control samples from the training set (GSE221521). (B) Boxplots of differential immune cells between control and DR groups (GSE221521). Orange represents DR samples, while blue represents control samples; statistical significance was determined by the Wilcoxon test (*p*  < 0.05). (C) Heatmap of Spearman correlation coefficients between the two biomarkers (SLC36A1 and RAB23) and differential immune cells (GSE221521). Color gradients represent the strength and direction of correlations, with significance markers defined as:  ^∗^ *p*  < 0.05,  ^∗∗^ *p*  < 0.01,  ^∗∗∗^ *p*  < 0.001, and  ^∗∗∗∗^ *p*  < 0.0001.

**Figure:**
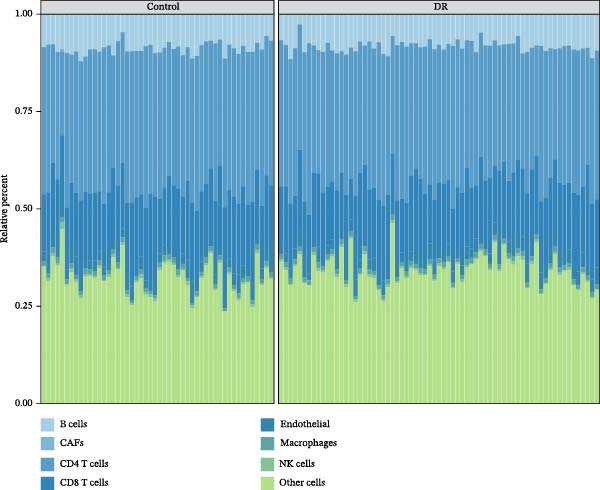
(A)

**Figure:**
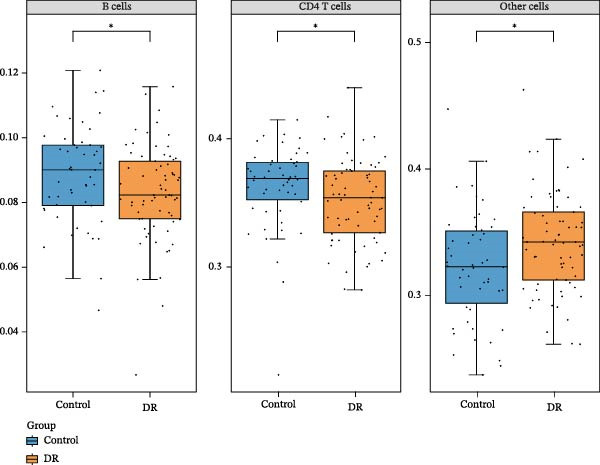
(B)

**Figure:**
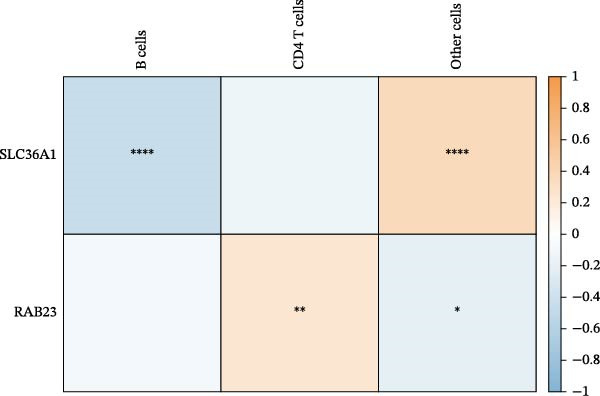
(C)

### 3.6. Three Networks of Biomarkers Were Constructed

Next, the GGI network revealed that 20 genes interacted with RAB23 and SLC36A1 (Figure 5A). Among these, EV15L, SMO, and SLC36A4 exhibited strong interactions with the biomarkers and other genes. Physical interactions were the most prevalent, and the genes in the network were primarily involved in amino acid transmembrane transport. In total, 856 miRNAs from miRWalk and 77 miRNAs from miRTarBase were intersected, yielding 19 miRNAs associated with the biomarkers (Figure S5A). These 19 miRNAs predicted 95 miRNA–lncRNA pairs from ENCORI and 136 from miRNet, resulting in 34 lncRNAs after intersection (Figure S5B). Based on these biomarkers, miRNAs, and lncRNAs, a ceRNA regulatory network was constructed (Figure 5B). The miRNAs most strongly associated with the regulation of biomarkers and lncRNAs were hsa‐miR‐16‐5p, hsa‐miR‐103a‐3p, hsa‐miR‐424‐5p, hsa‐miR‐15b‐5p, and hsa‐miR‐6838‐5p. Subsequently, 20 TFs were identified through the NetworkAnalyst platform, and a TF‐mRNA network was built, linking seven TFs to SLC36A1 and 14 TFs to RAB23 (Figure 5C). Notably, the TF “MBD1” was associated with both biomarkers. Finally, 15 drugs were predicted, including five targeting SLC36A1 and 11 targeting RAB23 (Figure 5D). Among these, methyl methanesulfonate was found to target both biomarkers, suggesting its potential as an effective treatment for DR.

Figure 5(A) GeneMANIA co‐expression network of SLC36A1 and RAB23. Nodes represent genes, and edges represent different types of interactions (physical, co‐expression, pathway, etc.). (B) ceRNA regulatory network, consisting of biomarkers (red nodes), miRNAs (green nodes), and lncRNAs (pink nodes). Edges represent regulatory interactions. (C) TF‐mRNA regulatory network, with TFs regulating the expression of SLC36A1 and RAB23 (red nodes). (D) Drug–biomarker network, showing potential drugs (blue nodes) targeting SLC36A1 and RAB23 (red nodes).

**Figure:**
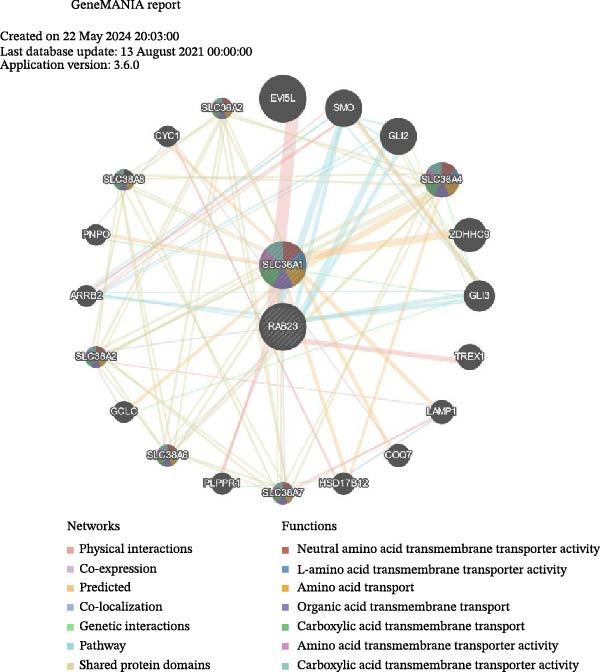
(A)

**Figure:**
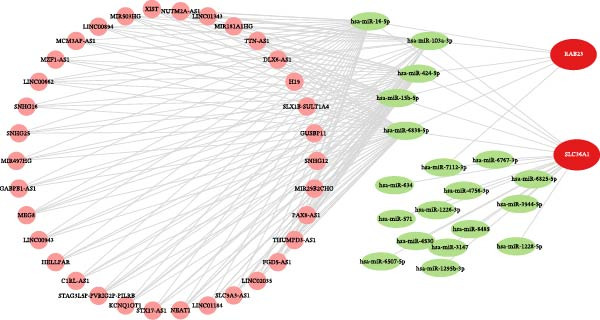
(B)

**Figure:**
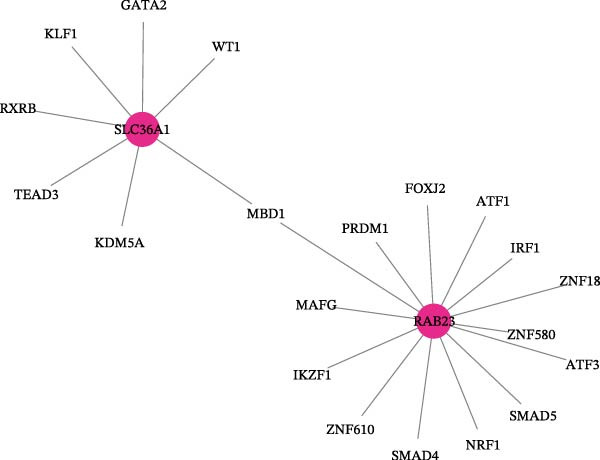
(C)

**Figure:**
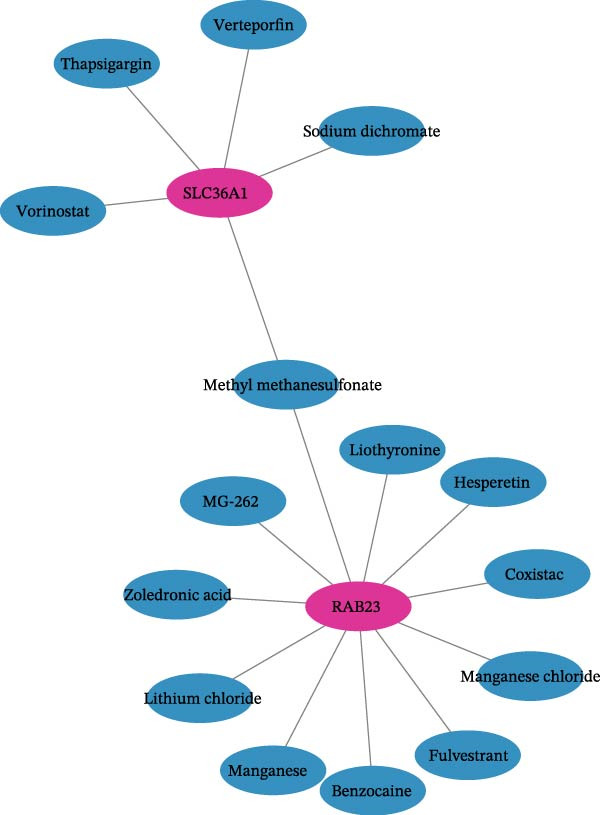
(D)

### 3.7. Dimensionality Reduction and Clustering of DR Single‐Cell Data

The raw data from the “GSE248284” dataset underwent QC (Figure S6A), resulting in 21,738 cells and 18,246 genes (Figure S6B). The top 2000 highly variable genes were identified for further analysis, with the top 10 most variable genes, including S100A9, S100A8, CST3, JCHAIN, IGKC, LYPD2, PPBP, AL928768.3, FCER1A, and PTGDS (Figure S6C). PCA revealed that the samples could be clustered into three groups (Figure S6D), with the significance diminishing after PC = 20, indicating that the first 20 principal components contained most of the sample information (Figure S6E). The curve plateaued at PC = 20 in the principal component fragmentation graph, leading to the selection of 20 principal components for further analyses (Figure S6F). UMAP clustering categorized the cells into 20 distinct cell clusters (Figure S6G).

### 3.8. B Cells and Classical Monocytes Were Identified as Key Cells

Based on the 20 cell clusters obtained, 4735 differentially expressed markers in these clusters were identified, and the top 3 expressed markers in each cluster were plotted (Figure S7A). Using markers extracted from a previous study [32] (Table 2), 11 cell clusters were annotated into 11 distinct cell types (Figure 6A). The expression levels of these markers across the 11 cell types are shown in Figure S7B, and the proportions of these cell types in DR samples are presented in Figure 6B, with T cells (33.03%) and classical monocytes (18.90%) making up the largest percentages. Figure 6C illustrates the distribution of these 11 cell types in the GSE248284 samples. Subsequently, 1266 differential markers were identified using the “FindAllMarkers” function and the Wilcoxon test. The expression levels of the top 20 differential markers in each cell type are plotted in Figure S7C. A total of 1365 GO entries were enriched by these markers, including terms related to mononuclear cell differentiation, leukocyte‐mediated immunity, and positive regulation of cytokine production (Figure S7D). These terms included 1170 BPs, 101 CCs, and 94 MFs, highlighting the critical roles of mononuclear cells and leukocytes in DR. Consistent with the hypothesis‐generating goal of this study, we identified B cells and classical monocytes as key cellular contexts for SLC36A1 and RAB23, based on their highest expression levels of these candidates, which provides a focused direction for future functional exploration rather than confirming a definitive functional role (Figure 6D). This criterion is valid for hypothesis generation, as it prioritizes cell types where the biomarkers are most likely to exert their biological effects, providing a focused direction for future functional validation. Thus, B cells and classical monocytes, which exhibited the highest expression levels of these biomarkers, were identified as key cells (Figure 6E) and selected for further analysis.

Figure 6(A) UMAP plot of cell cluster annotation, with 11 distinct cell types identified based on marker gene expression. (B) Pie chart showing the proportion of each cell type in DR samples (GSE248284), with T cells (33.03%) and classical monocytes (18.90%) being the most abundant. (C) Stacked bar plot showing the proportion of cell types in each individual DR sample (GSE248284), demonstrating inter‐sample heterogeneity in cellular composition. (D) UMAP plot of biomarker (SLC36A1 and RAB23) distribution in DR samples (GSE248284), with color intensity representing biomarker expression level. (E) Violin plots of biomarker expression levels between different cell clusters (GSE248284), showing that B cells and classical monocytes exhibit the highest expression of SLC36A1 and RAB23.

**Figure:**
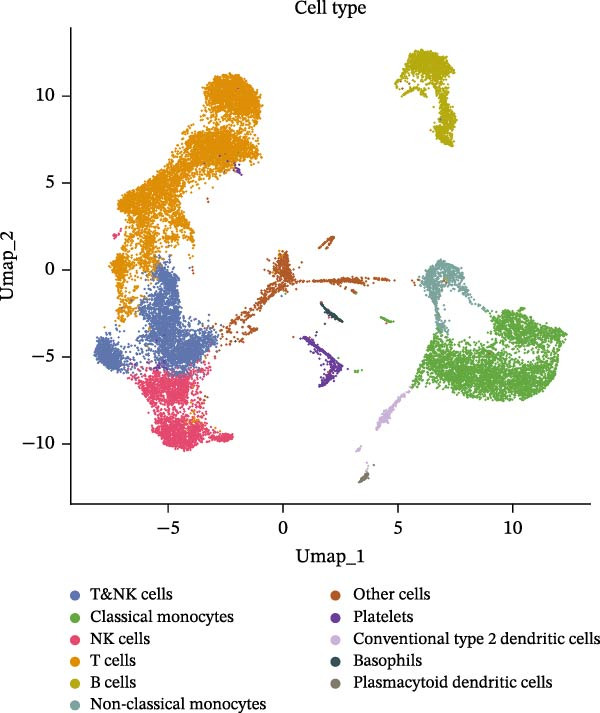
(A)

**Figure:**
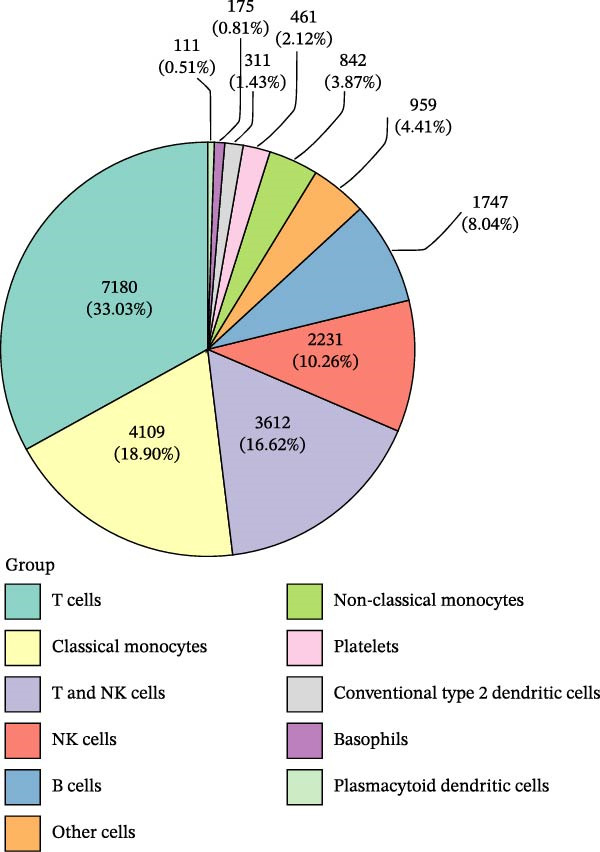
(B)

**Figure:**
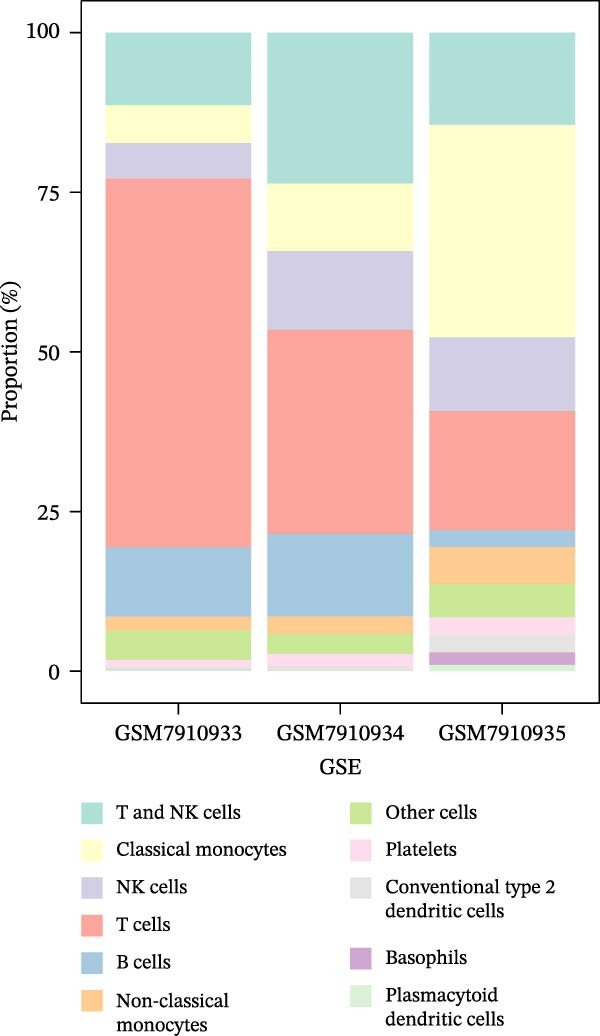
(C)

**Figure:**
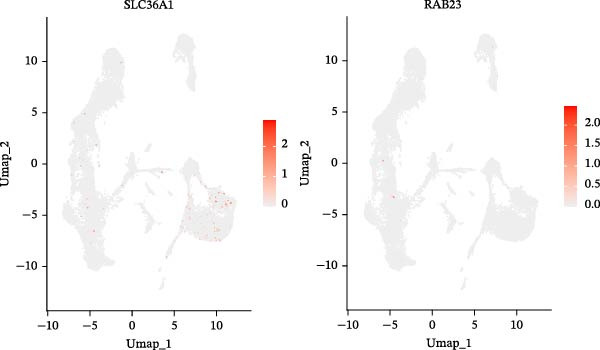
(D)

**Figure:**
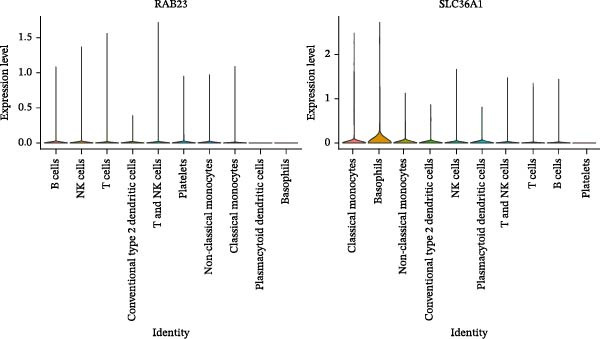
(E)

**Table 2:** Cell marker genes.

Cell type	Markers
T cells	CD3D, CCL5
NK cells	KLRC1, KLRF1, NKG7, KLRD1
Classical monocytes	CD14, FCN1, VCAN
Non‐classical monocytes	FCGR3A, IFITM3
B cells	MS4A1, CD79A
Conventional type 2 dendritic cells	CD1C, CD1E, FCER1A
Basophils	CLC, GATA2, MS4A3, MS4A2
Plasmacytoid dendritic cells	IL3RA, CLEC4C, LILRB4, GZMB
Platelets	PPBP, PF4, TUBB1, GP9
T cells and NK cells	Both T cells’ and NK cells’ genes
Other cells	—

### 3.9. Cell–Cell Communication Networks Constructed

From the cell–cell communication networks, T cells and classical monocytes were found to interact most frequently with other cells, indicating their complex synergistic role in DR. Another key cell type, B cells, primarily interacted with conventional type 2 dendritic cells and classical and non‐classical monocytes (Figure 7A–C). The cell–cell communication analysis also revealed the receptor–ligand pairs of key cells in terms of ligands (Figure 7D) and receptors (Figure 7E).

Figure 7(A, B) Cell communication interaction diagram. The size of the colored circles represents the number of cells: larger circles indicate a greater number of cells. The cells emitting arrows express ligands, while the cells pointed to by the arrows express receptors. More ligand–receptor pairs result in thicker lines. (C) Heatmap of the number of interactions between different cell clusters in DR samples. Color intensity represents the total count of ligand–receptor interactions between each pair of cell clusters, with white squares indicating no significant interaction detected. (D, E) Key ligand–receptor pairs mediating communication from key cells to other cell types in DR samples.

**Figure:**
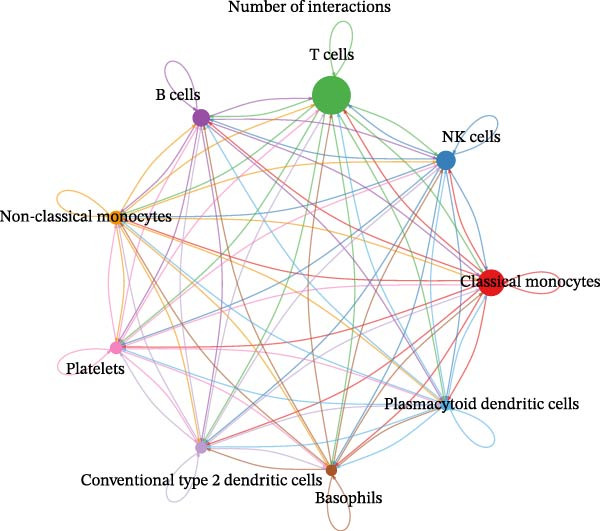
(A)

**Figure:**
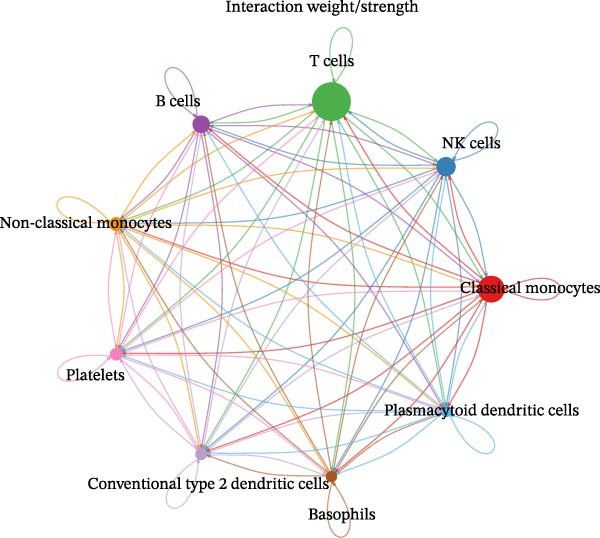
(B)

**Figure:**
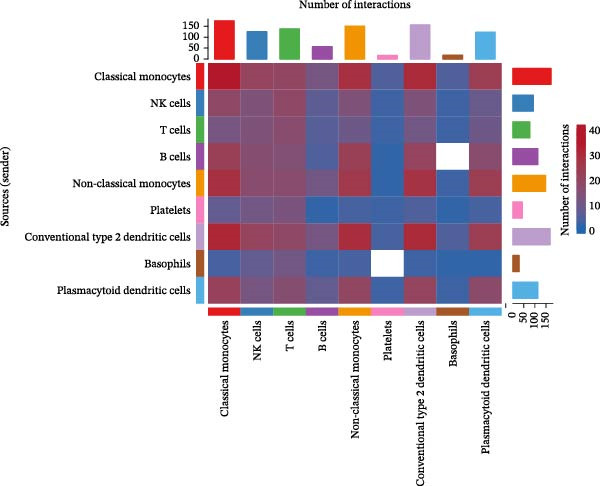
(C)

**Figure:**
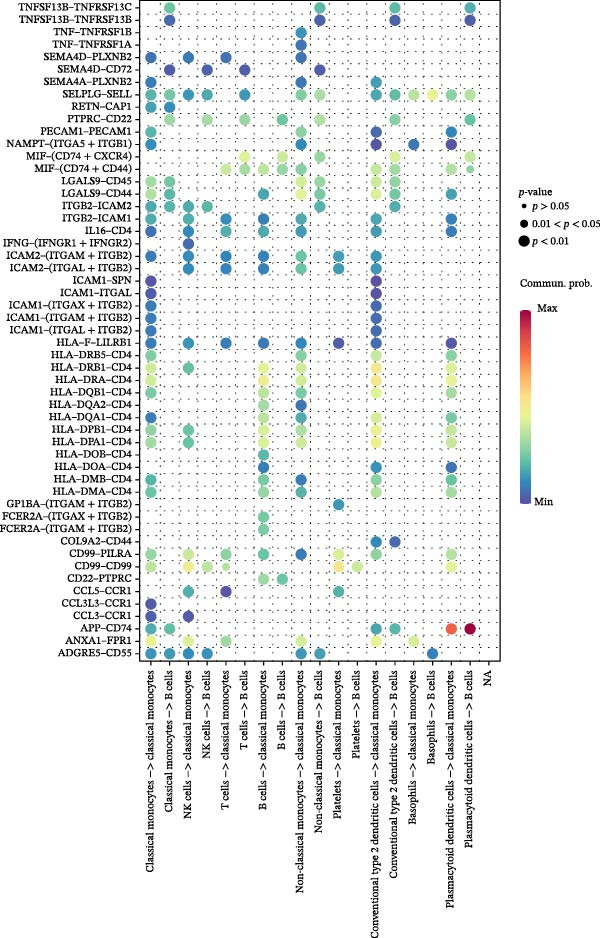
(D)

**Figure:**
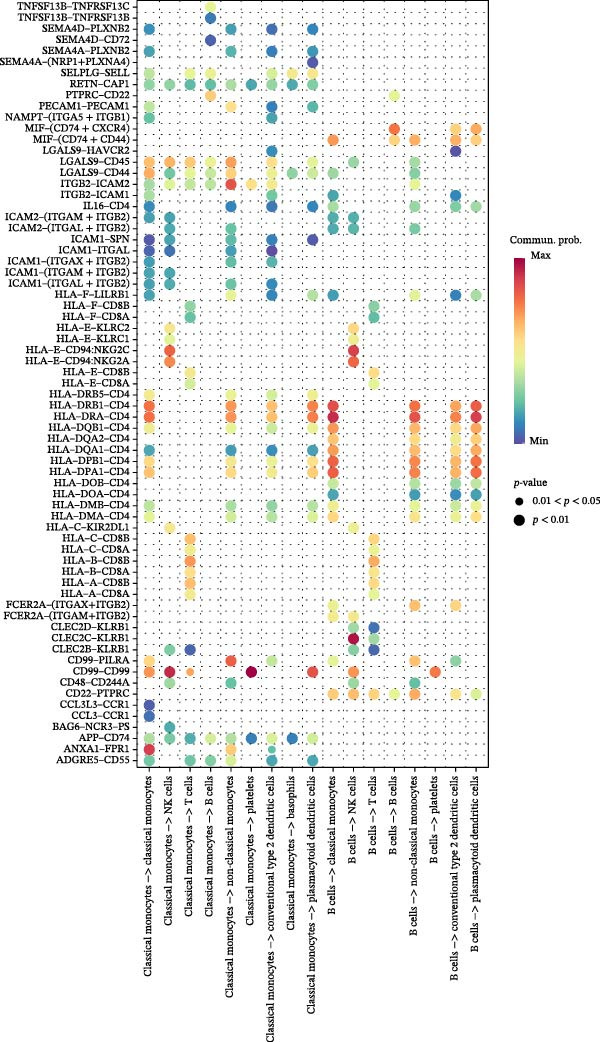
(E)

### 3.10. Pseudotime Trajectories and Biomarkers’ Expression in Different Developmental Stages of B Cells and Classical Monocytes

Before pseudotime analysis, B cells and classical monocytes were clustered into 4 and 5 subgroups, respectively, using dimensionality reduction (Figure 8A,B). The pseudotime trajectory of B cells revealed that these cells branched into 9 distinct developmental stages, with subgroup 3 predominantly in stage 1 and subgroup 2 predominantly in stage 7 (Figure 8C–E). Classical monocytes were divided into five developmental stages, with subgroup 3 predominantly in stage 1 and subgroups 2 and 4 predominantly in stage 4 (Figure 8F–H). The expression patterns of biomarkers RAB23 and SLC36A1 at different stages of B cell (Figure 8I) and classical monocyte (Figure 8J) development showed that RAB23 maintained a consistent expression level across both cell types, while SLC36A1’s expression was high in the early stages and gradually declined until it stabilized. Pseudotime analysis reconstructed the developmental trajectories of both B cells and classical monocytes, suggesting dynamic transitions in cell states during DR. Notably, SLC36A1 exhibited a consistent high‐to‐low expression pattern from the early to terminal stages in both cell types, indicating a stage‐specific functional contribution.

Figure 8(A, B) UMAP clustering results of key cell subgroups; the analysis was performed using PCA and UMAP dimensionality reduction. (C–E) Pseudotime trajectory plots of B cells. (F–H) Pseudotime trajectory plots of classical monocytes. (I, J) Expression trend plots of biomarkers (RAB23 and SLC36A1) across different developmental stages of key cells. The *x*‐axis represents pseudotime, the *y*‐axis represents relative expression level.

**Figure:**
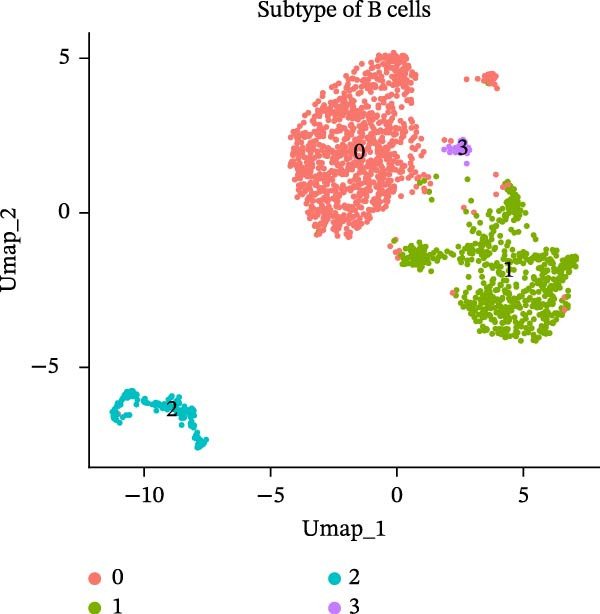
(A)

**Figure:**
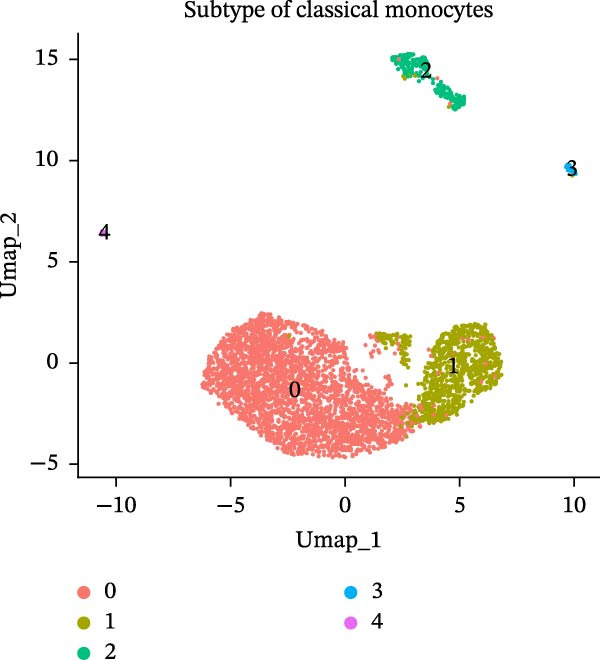
(B)

**Figure:**
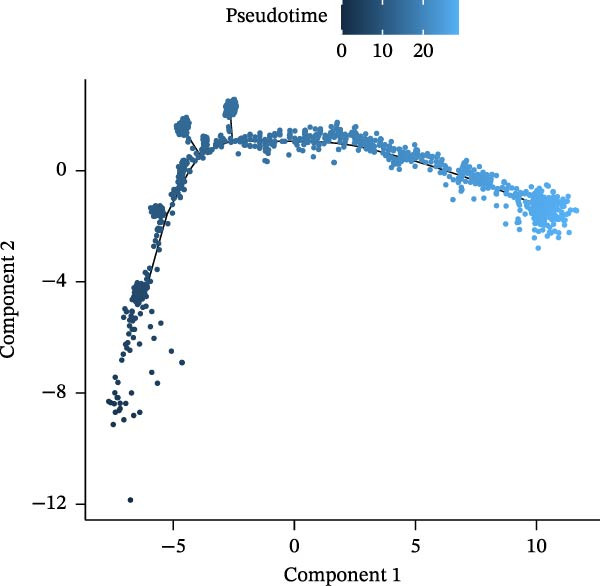
(C)

**Figure:**
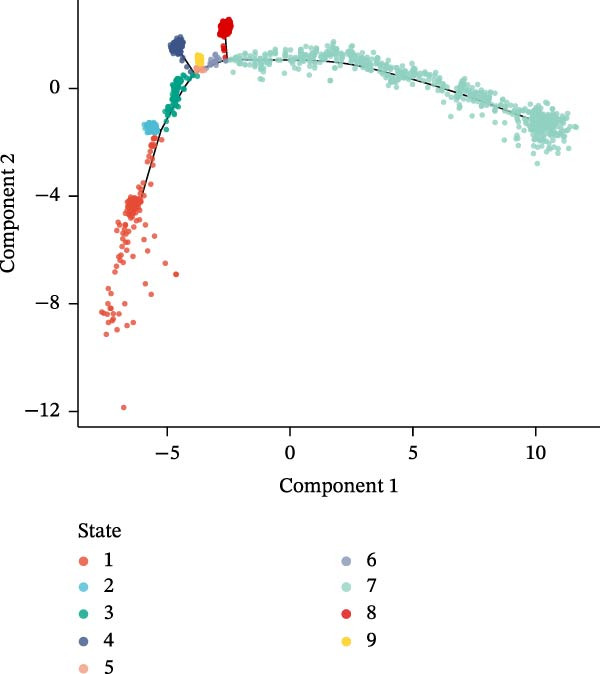
(D)

**Figure:**
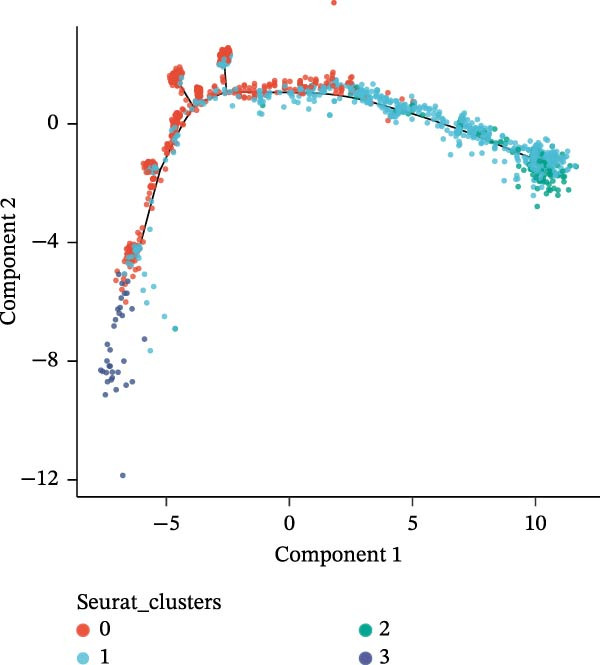
(E)

**Figure:**
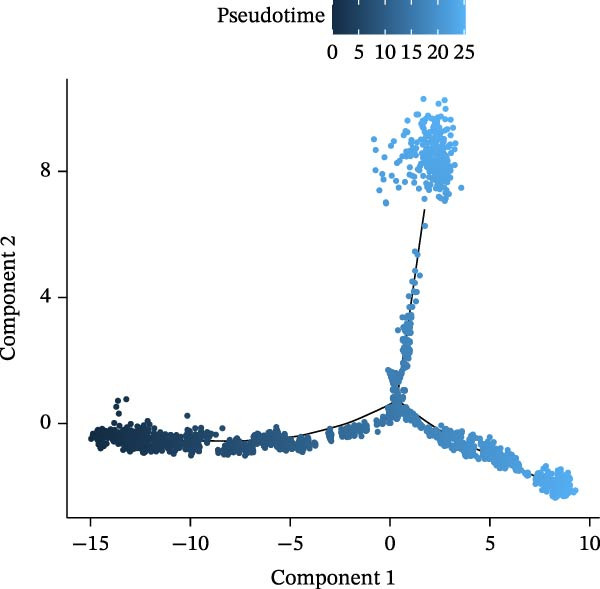
(F)

**Figure:**
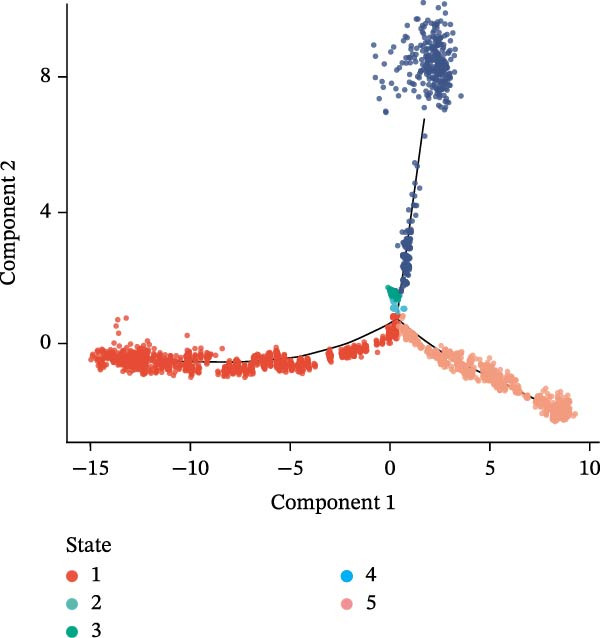
(G)

**Figure:**
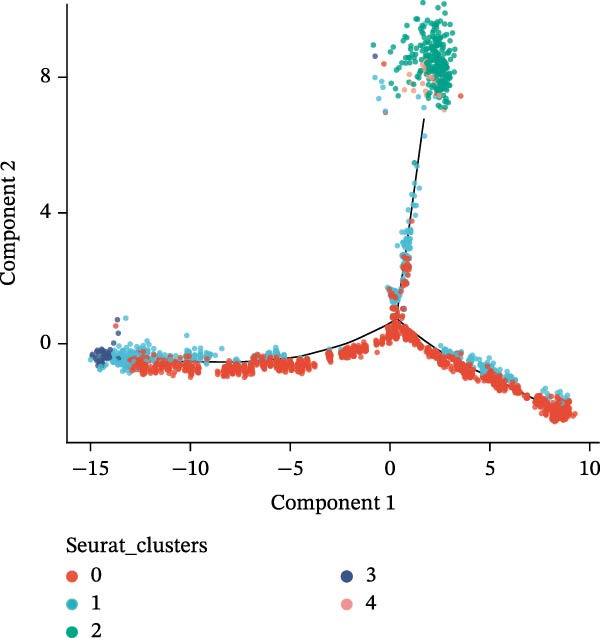
(H)

**Figure:**
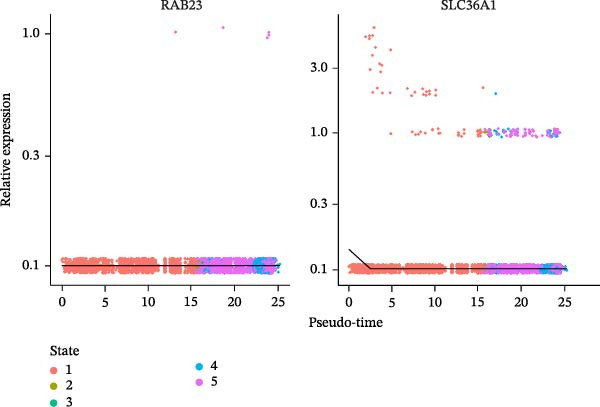
(I)

**Figure:**
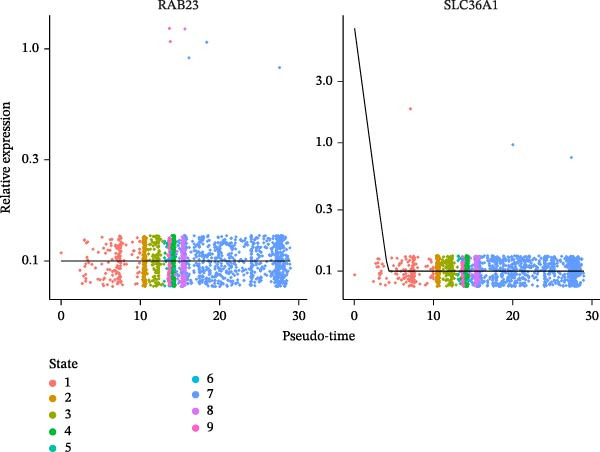
(J)

### 3.11. Result of RT‐qPCR Validation

The expression levels of RAB23 and SLC36A1 were further evaluated in clinical samples. RT‑qPCR analysis revealed a significant upregulation of SLC36A1 and RAB23 in the DR group compared with controls (*F* = 5.184 *p* = 0.027 < 0.05; *F* = 4.147 *p* = 0.047 < 0.05). (Figure 9A,B). During the preliminary validation stage, we also examined an additional dataset GSE185011, which showed divergent expression trends for these two biomarkers relative to our training set (GSE221521), validation set (GSE189005), and clinical RT‑qPCR results. Due to this inconsistency and potential cohort heterogeneity, GSE185011 was excluded from the formal analysis to ensure the robustness of the present study. Our current results based on two consistent transcriptomic datasets and experimental validation support the upregulation of SLC36A1 and RAB23 in DR.

Figure 9(A) Relative mRNA expression of SLC36A1 in control (black) and DR (gray) groups, normalized to β‐actin. (B) Relative mRNA expression of RAB23 in control (black) and DR (gray) groups, normalized to β‐actin. Significance markers: ^∗^ *p*  < 0.05.

**Figure:**
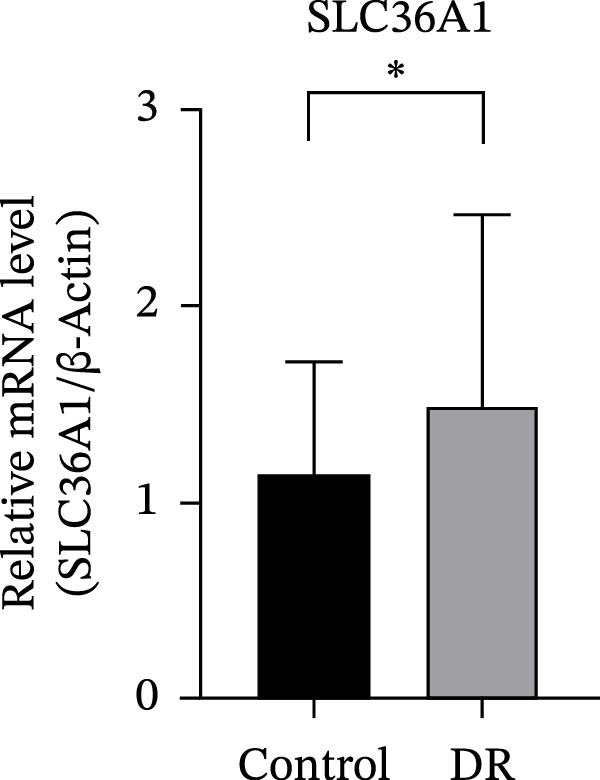
(A)

**Figure:**
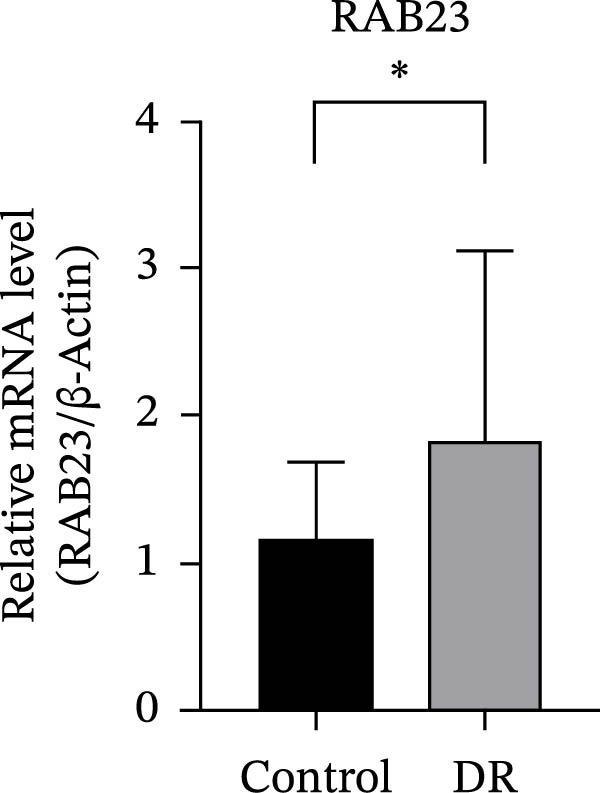
(B)

## 4. Discussions

DR is a common chronic complication of diabetes and a progressive disease that can impair vision and lead to blindness [3]. VD has been shown to alleviate DR or reduce its incidence by decreasing oxidative stress, regulating inflammation and immune responses, reducing endoplasmic reticulum stress, and controlling endothelial cell apoptosis [11]. However, the mechanisms of action and diagnostic potential of VDRGs remain unexplored. Thus, further investigation into the relationship between VD metabolism and DR is essential.

Our study nominates RAB23 as a potential downstream candidate associated with VD signaling in DR pathogenesis. RAB23 is a small GTPase from the Rab family, involved in the formation, transport, and fusion of vesicles during the autophagic process [35, 36]. Autophagy is closely associated with DR development [37], as it promotes endothelial cell proliferation and migration under high‐glucose conditions, both of which are critical for neovascularization in DR [38]. Moderate autophagy protects retinal vascular endothelial cells, pericytes, and pigment epithelial cells in high‐glucose environments, while insufficient or excessive autophagy may contribute to the onset and neurodegenerative changes seen in DR [39, 40]. Our VD‐centered analysis provides novel insights into the potential interaction between VD signaling and RAB23‐mediated autophagy. Given that VD has been reported to modulate autophagic activity in various cellular contexts [41, 42], dysregulated RAB23 in DR may reflect impaired VD signaling, disrupting protective autophagy and contributing to disease progression.

Additionally, RAB23 enhances PDGFRα signaling to activate ERK1/2, which in turn regulates the expression of N‐cadherin [43]. N‐cadherin plays a significant role in the proliferation, migration, and invasiveness of retinal Müller cells and pigment epithelial cells under high‐glucose conditions, which are key in the formation of fibrovascular membranes in proliferative DR [44, 45]. N‐cadherin also maintains tight junctions between endothelial cells and pericytes, supporting retinal barrier integrity, vascular permeability, and microvascular stability [46]. Therefore, it can be inferred that RAB23, by regulating N‐cadherin expression, may promote the onset and progression of DR.

Our integrated analysis also identified SLC36A1 as a novel downstream modulator connecting VD status to mTORC1 signaling in DR. SLC36A1, also known as proton‐coupled amino acid transporter 1 (PAT1), is an amino acid transporter that plays a key role in mTORC1 activation [47]. mTOR exists as two distinct protein complexes—mTORC1 and mTORC2—each with unique physiological functions. mTORC1 regulates cellular metabolism by promoting protein synthesis and inhibiting autophagy [48–51]. Since proper autophagy has protective effects on retinal vascular endothelial cells, pericytes, and pigment epithelial cells under high‐glucose conditions [39, 40], the activation of mTORC1 is linked to the onset and progression of DR. Furthermore, in a diabetic rat model, mTORC1 activation leads to the production of p‐S6 protein, which regulates the expression of VEGF and PEDF, thereby influencing endothelial cell proliferation and migration—key features of DR [52]. DR is characterized not only by microvascular changes but also by neurodegenerative alterations, associated with the activation and proliferation of retinal Müller cells following injury [53]. Retinal Müller cells provide structural and neurotrophic support between neurons and vessels and regulate retinal neurotransmitter homeostasis [54]. The PPP1CA/YAP/GS/Gln/mTORC1 signaling pathway activates retinal Müller cells during DR, leading to retinal ganglion cell apoptosis [55]. This highlights the significant role of mTORC1 in DR. The novel insight from our study is the potential connection between mTORC1 activation and VD status. Given that VD regulates both mTOR and amino acid metabolism, SLC36A1 may serve as a functional mediator through which VD deficiency (or impaired signaling) influences mTORC1 activation in the retinal microenvironment, thereby contributing to the vascular and neurodegenerative changes seen in DR.

Moreover, SLC36A1 is primarily involved in amino acid transport, facilitating the uptake of various amino acids and dipeptides, including glycine [56]. Amino acid metabolism is implicated in diabetes development, with studies showing that glycine supplementation can significantly reduce blood glucose levels [57]. Additionally, alterations in branched‐chain amino acid (BCAA) concentrations may affect insulin sensitivity and glycemic control by influencing lipid metabolism and inflammatory responses [4]. Given that unstable glycemia is linked to DR severity [58], it is possible that SLC36A1 may influence the onset and progression of DR by transporting specific amino acids.

Chronic retinal inflammation caused by hyperglycemia plays a significant role in the development of DR [4]. Elevated levels of pro‐inflammatory cytokines, including TNF‐α, IL‐8, MCP‐1, IL‐6, IL‐26, and IL‐1β, have been detected in the vitreous of patients with DR [59]. In contrast, B cells are known to secrete the anti‐inflammatory cytokine IL‐10 [60]. Enhancing miR‐19a expression and histone deacetylase (HDAC) activity in patients with DR, which suppresses IL‐10 expression in peripheral B cells, may exacerbate the inflammatory processes of DR [61]. Additionally, B cells may contribute to the immune response in DR by producing immunoglobulins such as IgA and IgM [62]. Our study further highlighted the role of B cells as key immune cells in DR, revealing differences in both immune infiltration analysis and single‐cell analysis. In comparison to the normal control group, DR samples exhibited fewer B cells, and SLC36A1 expression was negatively correlated with B cell levels, suggesting higher SLC36A1 expression in DR. Notably, while T cells were the most abundant cell type (33.03%) in DR samples, B cells and classical monocytes were selected as key cells based on their highest expression of SLC36A1 and RAB23—consistent with our hypothesis‐generating goal of identifying the most likely cellular “actors” for the biomarkers’ functions. This distinction is biologically meaningful: T cells are widely recognized as central mediators of adaptive immunity in DR, but their high abundance does not necessarily indicate they are the primary cell types mediating the VD‐related effects of SLC36A1 and RAB23. In contrast, B cells (key producers of anti‐inflammatory cytokines like IL‐10) and classical monocytes (critical for initiating and regulating inflammatory responses in retinal injury) are functionally relevant to the pathological processes of DR (e.g., chronic inflammation and vascular damage) that our biomarkers are linked to [61, 63]. Their high expression of SLC36A1 and RAB23 suggests these cells may be the primary targets through which VD metabolism influences DR progression, whereas T cells, despite their abundance, may play more general immune roles unrelated to the specific VD‐biomarker axis explored in this study. Pseudotime analysis of B cell development revealed an “L”‐shaped expression trend for SLC36A1, with high expression in the early stages, followed by a gradual decline until stabilization. This indicates that SLC36A1 expression is a dynamic process that varies with the developmental stage, and the results could differ depending on the time point of analysis. A significant upregulation in the expression of RAB23 or SLC36A1 was observed between the DR and control groups, which aligns with findings from the GSE221521 and GSE189005 datasets.

Regulatory network analysis indicated that both SLC36A1 and RAB23 are regulated by the ceRNA network, where their expression may be jointly controlled by multiple miRNAs, lncRNAs, and TFs. One such miRNA, miRNA‐16‐5p, is associated with the biomarkers. miRNA‐16‐5p inhibits cell proliferation and angiogenesis in colorectal cancer by negatively regulating FOXK1 and blocking the PI3K/Akt/mTOR pathway [64]. Aberrant activation of the PI3K/Akt/mTOR pathway has been implicated in DR progression, and inhibiting this pathway could restore autophagy function, alleviating retinopathy [65, 66]. MEG8, an lncRNA associated with the biomarkers, has been reported to be upregulated in high‐glucose environments and induce cell apoptosis by regulating miR‐770‐5p through methylation. This mechanism may contribute to the development of diabetic nephropathy [67]. However, the roles of the miRNAs, lncRNAs, and TFs associated with the biomarkers, as identified through database screening, have not been explored in the context of DR and warrant further investigation.

In this study, module genes were identified using ssGSEA and WGCNA, and candidate genes SLC36A1 and RAB23 were selected via machine learning. Combined with single‐cell transcriptome analysis, we systematically explored the potential roles of VD signaling‑related genes in DR. The novelty of this work is not the discovery of SLC36A1 and RAB23 themselves, but their identification as VD network‑associated candidates using a VD‑focused bioinformatics pipeline. These two genes are not canonical components of the VD metabolic pathway. Instead, they were identified as key candidates because their expression strongly correlated with VD‑related gene modules. They may represent potential downstream components rather than established effectors or regulatory nodes in a broader VD‑associated network, potentially acting through autophagy and mTOR signaling. The relationship between VD signaling and these candidates is inferred from co‑expression and computational analyses, not from direct experimental evidence of regulation. Thus, while our findings expand the current understanding of VD in DR and provide new directions for functional research, this proposed VD‑SLC36A1/RAB23 axis requires further experimental validation.

## 5. Limitations

Despite the rigor of the bioinformatics approach, several limitations should be acknowledged. First, this study is designed as a hypothesis‐generating, systems biology investigation rather than a definitive functional study. The association between VD signaling and the identified biomarkers SLC36A1 and RAB23 is correlative rather than causative, and no direct mechanistic evidence supports a regulatory or causal link at present. Second, the absence of direct functional evidence from in vitro cellular or in vivo animal models means the precise biological roles and regulatory mechanisms of SLC36A1 and RAB23 in DR pathogenesis remain to be further validated. Third, although our RT‐qPCR results were consistent with the transcriptomic datasets, the sample size for RT‐qPCR (*n* = 24 per group) is still modest, which reduces the statistical power and robustness of the conclusions. Fourth, the single‐cell RNA‐seq dataset lacks detailed clinical metadata such as disease stage, diabetes duration, and treatment status. Unknown clinical confounders may contribute to sample heterogeneity and influence the stability of cell‐type‐specific conclusions. Fifth, the constructed ceRNA network, TF–mRNA regulatory network, and drug prediction results are all purely predictive based on public databases and computational algorithms. Their actual regulatory relationships and therapeutic effects have not been verified by experiments. During preliminary validation, the external dataset GSE185011 exhibited inconsistent expression trends of SLC36A1 and RAB23 compared with our main datasets and experimental results. This discrepancy likely reflects substantial heterogeneity among DR cohorts, including differences in sample source, disease stage, sequencing platform, and patient population. The exclusion of GSE185011 due to inconsistent expression trends further underscores the need for validation in larger, multicenter cohorts with standardized clinical metadata to reduce the impact of cohort‐specific heterogeneity. Moreover, although single‐cell analysis localized biomarker expression in B cells and classical monocytes, these associations are observational, and the biological functions of SLC36A1 and RAB23 in these cell types require further investigation. Therefore, SLC36A1 and RAB23 are regarded as high‐confidence candidate biomarkers rather than definitive ones. Future research will focus on exploring their biological functions through in vitro and in vivo studies, and validating their expression and clinical utility in larger and multicenter cohorts is essential to confirm their reliability as diagnostic biomarkers for DR.

## 6. Conclusions

This study focused on VD signaling‑associated downstream candidate genes in DR. By integrating transcriptomic data from public databases and existing literature, and applying WGCNA and machine learning algorithms, SLC36A1 and RAB23 are nominated as high‑priority, VD‑network‑associated candidate genes rather than definitive biomarkers for DR. Functional enrichment analysis revealed that both candidates are closely linked to carbohydrate metabolism. Immune infiltration analysis identified B cells and CD4 T cells as differential immune cells between DR and control groups. GGI networks, ceRNA regulatory networks, and TF‑mRNA networks were constructed, highlighting that SLC36A1 and RAB23 may be regulated by multiple miRNAs, lncRNAs, and TFs. Additionally, 15 potential therapeutic agents for DR were predicted, offering directions for future drug development. Single‑cell analysis implicates B cells and classical monocytes as relevant cellular contexts for these candidate genes and reveals candidate expression changes at different developmental stages. RT‐qPCR experiments provided preliminary validation of the upregulation of SLC36A1 and RAB23 in the DR group, consistent with transcriptomic findings from GSE221521 and GSE189005. Overall, this integrated analysis nominates promising candidates and provides a framework for hypothesis generation, rather than establishing definitive biomarkers or mechanisms. This study does not establish SLC36A1 and RAB23 as definitive diagnostic biomarkers or confirm their causal role in DR; instead, it provides testable hypotheses and an analytical framework to guide future functional and clinical validation studies.

## Author Contributions

Yuhua Hao and Pengfei Chen developed the research design. Ruiqi Li and Keren Zhao recruited patients and conducted the study and performed the data acquisition. Rui Liu prepared figures and tables. All authors contributed in data analysis and interpretation and in manuscript preparation.

## Funding

This study was funded by the 2024 Government‐Sponsored Clinical Medicine Talent Training Program for Outstanding Talents, Project Number ZF2024097, the 2026 Hebei Provincial Medical Science Research Project Plan, Project Number 20260588, the Hebei Natural Science Foundation, Project Number H2025206588, and the Hebei Provincial Special Project for Joint Innovation among Medical Institutions, Research Institutes, and Enterprises, Project Number LH20250075.

## Disclosure

All authors have read and approved the final manuscript.

## Ethics Statement

This study was approved by the Research Ethics Committee of the Fourth Hospital of Hebei Medical University (2024KS100) and was conducted in accordance with the tenets of the Declaration of Helsinki.

## Consent

All participants had given their written informed consent to take part in this study.

## Conflicts of Interest

The authors declare no conflicts of interest.

## Supporting Information

Additional supporting information can be found online in the Supporting Information section.

## Supporting information


**Supporting Information** Figure S1. (A) Exclusion of discrete samples in WGCNA. (B) Retained samples after quality control. Figure S2. (A, B) ROC curve analysis of RAB23 and SLC36A1 in the training set GSE221521. (C, D) ROC curve analysis of RAB23 and SLC36A1 in the validation set GSE189005. Figure S3. (A) Spatial structure of the RAB23 protein. (B) Amino acid sequence of the RAB23 protein. (C) Spatial structure of the SLC36A1 protein. (D) Amino acid sequence of the SLC36A1 protein. (E) Subcellular localization of SLC36A1. (F) Subcellular localization of RAB23. (G) Diseases associated with the biomarkers. Figure S4. (A–F) Scatter plots showing correlations between biomarkers (SLC36A1 and RAB23) and differential immune cells. Figure S5. (A) Venn diagram of miRNAs predicted by miRTarBase and miRWalk databases. (B) Venn diagram of lncRNAs predicted by ENCORI and miRNet databases. Figure S6. (A) Distribution of nFeature_RNA and nCount_RNA before quality control. (B) Distribution of nFeature_RNA and nCount_RNA after quality control. (C) Highly variable gene selection. (D) PCA analysis of different samples. (E) Principal component line chart. (F) Scree plot of principal components. (G) UMAP clustering plot of cell clusters. Figure S7. (A) Bubble plot of marker gene expression levels. (B) Violin plots of specific marker gene expression in each cell type. (C) Heatmap of marker gene expression levels. (D) GO enrichment results of marker genes.

## Data Availability

All processed data matrices (differentially expressed gene lists, cell cluster markers, co‐expression modules, and biomarker annotation results) and the full bioinformatics analysis scripts used in this study are publicly available in a GitHub repository and permanently archived at Zenodo with the DOI: 10.5281/zenodo.18883388. Raw sequencing data were obtained from the GEO database under Accession Numbers GSE221521, GSE189005, and GSE248284.
