# Supplementary material for: Exploring Vitamin D Signaling‐Associated Biomarkers and Their Diagnostic Value in Diabetic Retinopathy: A Combined Transcriptomic and Single‐Cell Analysis With Experimental Validation
Source: J Diabetes Res. 2026 Apr 30;2026:7240252. doi: 10.1155/jdr/7240252 (PMC13129936; doi:10.1155/jdr/7240252)
Supplement: Supplementary file 1 — Supporting Information Figure S1. (A) Exclusion of discrete samples in WGCNA. (B) Retained samples after quality control. Figure S2. (A, B) ROC curve analysis of RAB23 and SLC36A1 in the training set GSE221521. (C, D) ROC curve analysis of RAB23 and SLC36A1 in the validation set GSE189005. Figure S3. (A) Spatial structure of the RAB23 protein. (B) Amino acid sequence of the RAB23 protein. (C) Spatial structure of the SLC36A1 protein. (D) Amino acid sequence of the SLC36A1 protein. (E) Subcellular localization of SLC36A1. (F) Subcellular localization of RAB23. (G) Diseases associated with the biomarkers. Figure S4. (A–F) Scatter plots showing correlations between biomarkers (SLC36A1 and RAB23) and differential immune cells. Figure S5. (A) Venn diagram of miRNAs predicted by miRTarBase and miRWalk databases. (B) Venn diagram of lncRNAs predicted by ENCORI and miRNet databases. Figure S6. (A) Distribution of nFeature_RNA and nCount_RNA before quality control. (B) Distribution of nFeature_RNA and nCount_RNA after quality control. (C) Highly variable gene selection. (D) PCA analysis of different samples. (E) Principal component line chart. (F) Scree plot of principal components. (G) UMAP clustering plot of cell clusters. Figure S7. (A) Bubble plot of marker gene expression levels. (B) Violin plots of specific marker gene expression in each cell type. (C) Heatmap of marker gene expression levels. (D) GO enrichment results of marker genes. [file JDR-2026-7240252-s001.docx]

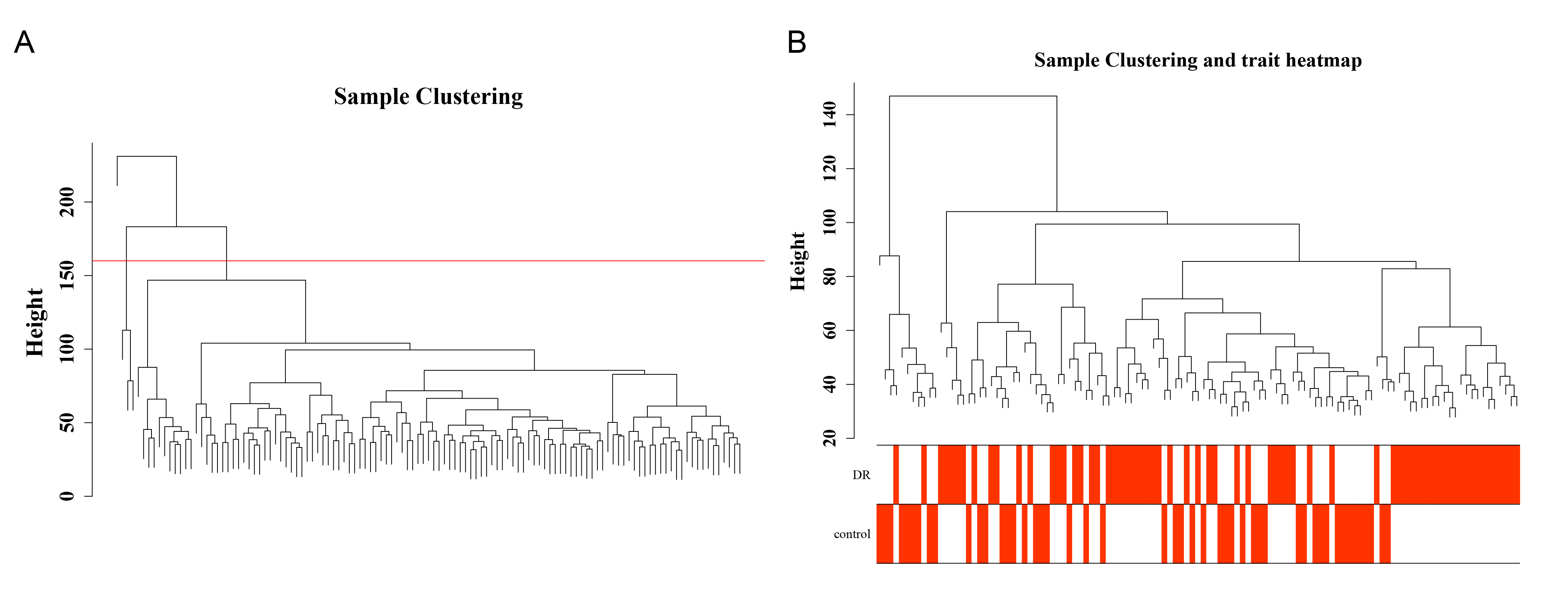
 **Figure S1.** (A) Exclusion of discrete samples in WGCNA; (B) Retained samples after quality control.


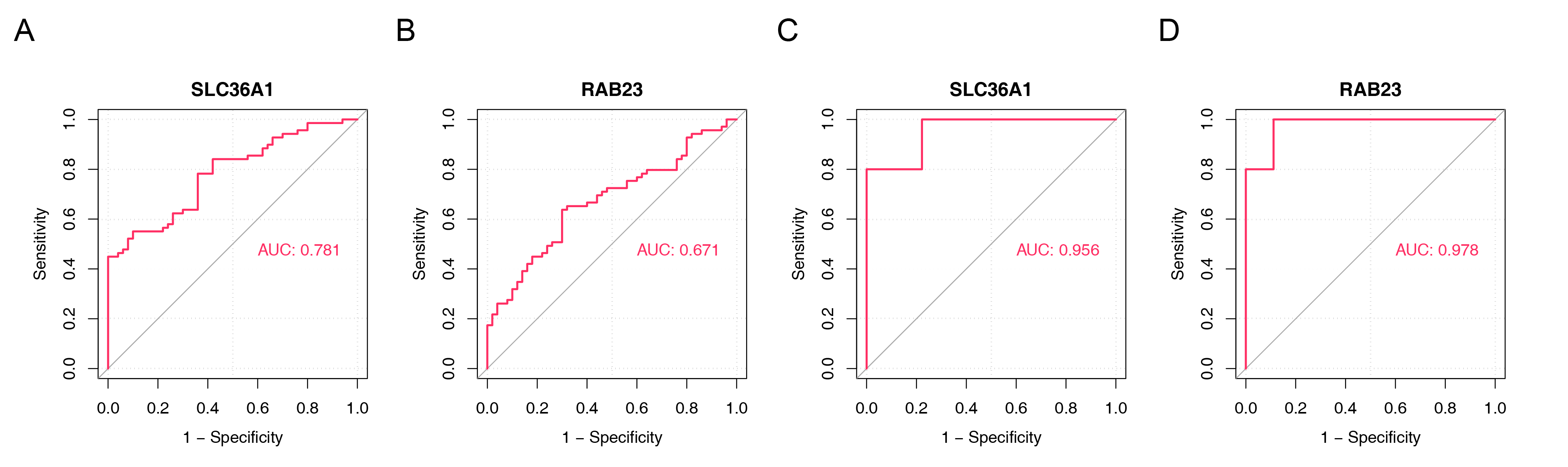


**Figure S2.** (A-B) ROC curve analysis of RAB23 and SLC36A1 in the training set GSE221521; (C-D) ROC curve analysis of RAB23 and SLC36A1 in the validation set GSE189005.


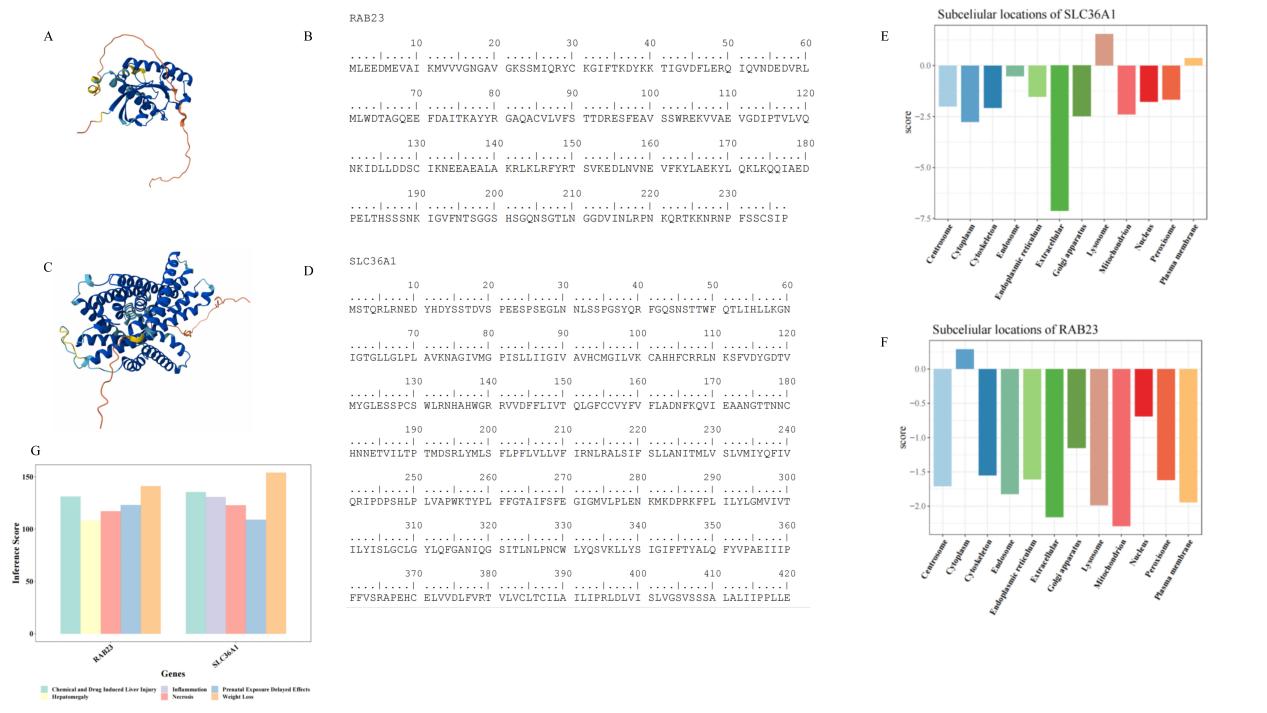


**Figure S3.** (A) Spatial structure of the RAB23 protein; (B) amino acid sequence of the RAB23 protein; (C) spatial structure of the SLC36A1 protein; (D) amino acid sequence of the SLC36A1 protein; (E) subcellular localization of SLC36A1; (F) subcellular localization of RAB23; (G) diseases associated with the biomarkers.


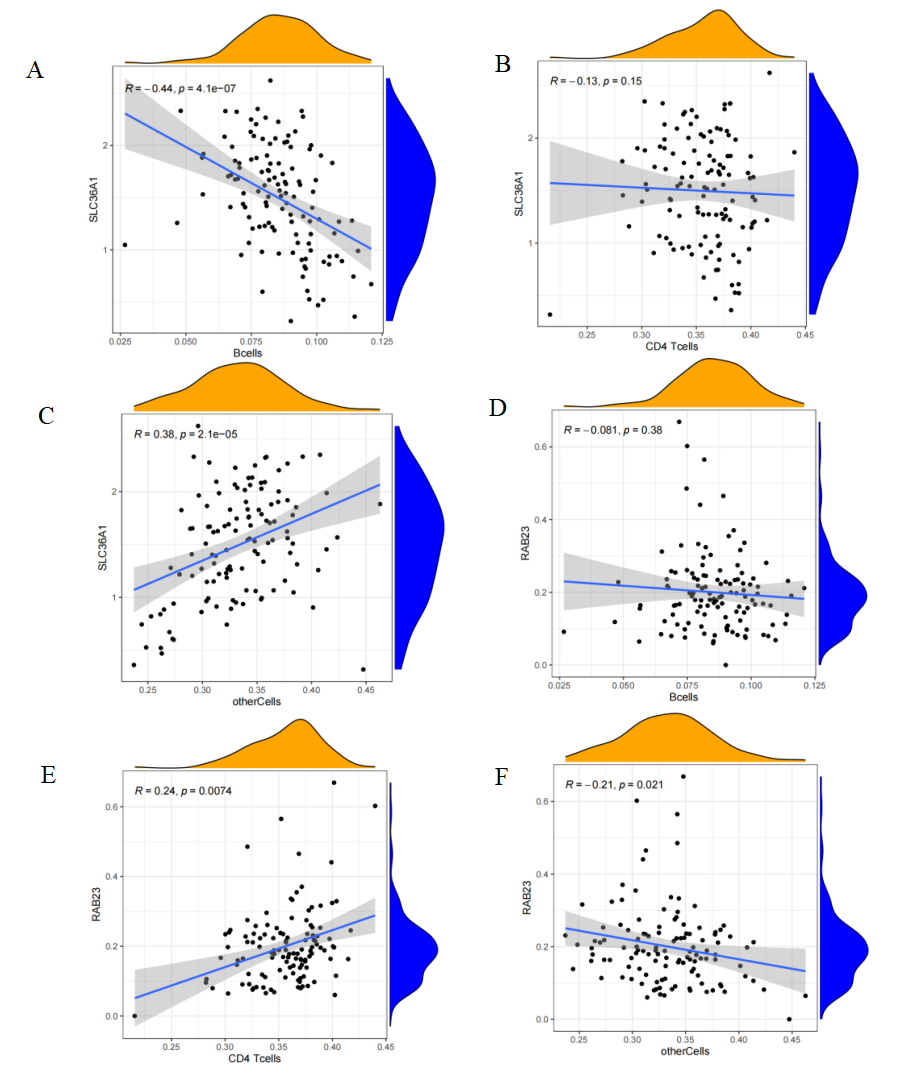


**Figure S4.** (A-F) Scatter plots showing correlations between biomarkers (SLC36A1 and RAB23) and differential immune cells.


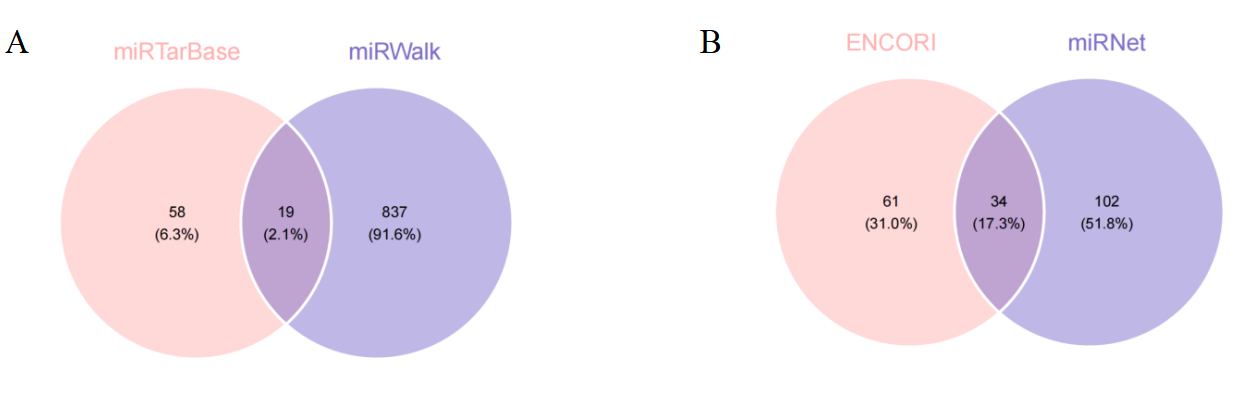


**Figure S5.** (A) Venn diagram of miRNAs predicted by miRTarBase and miRWalk databases; (B) Venn diagram of lncRNAs predicted by ENCORI and miRNet databases.


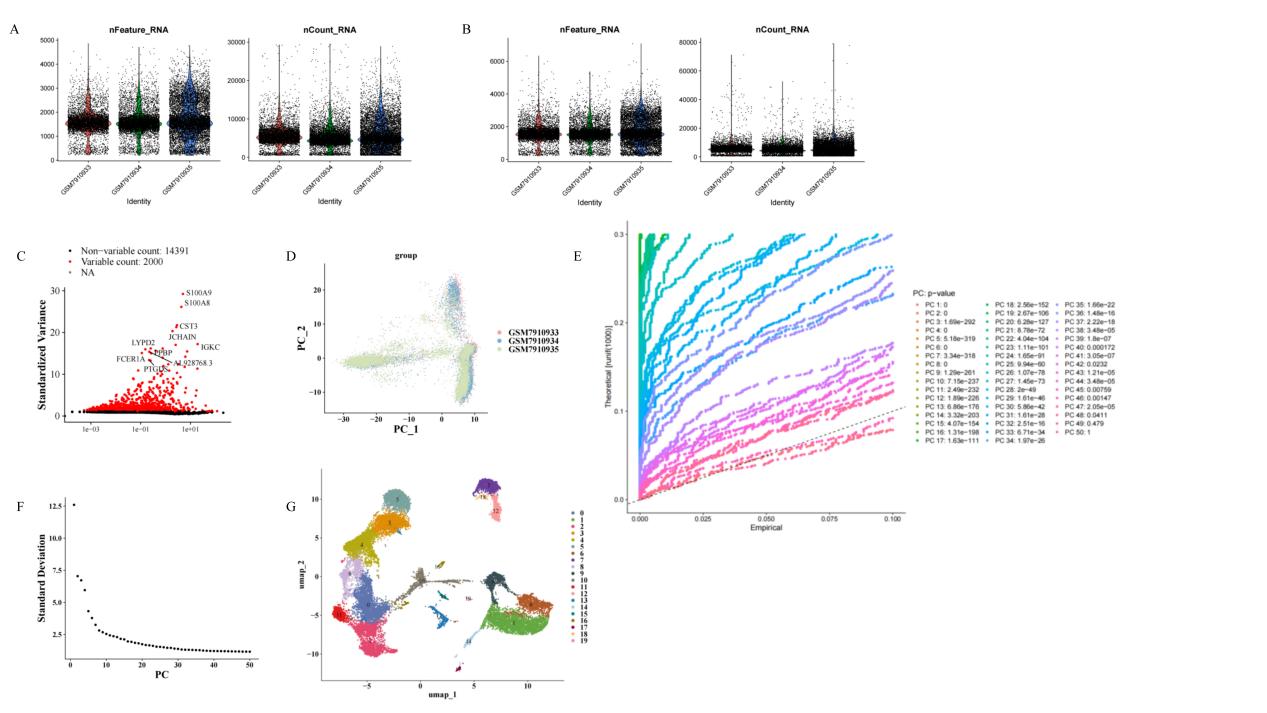


**Figure S6.** (A) Distribution of nFeature_RNA and nCount_RNA before quality control; (B) distribution of nFeature_RNA and nCount_RNA after quality control; (C) highly variable gene selection; (D) PCA analysis of different samples; (E) principal component line chart; (F) scree plot of principal components; (G) UMAP clustering plot of cell clusters.


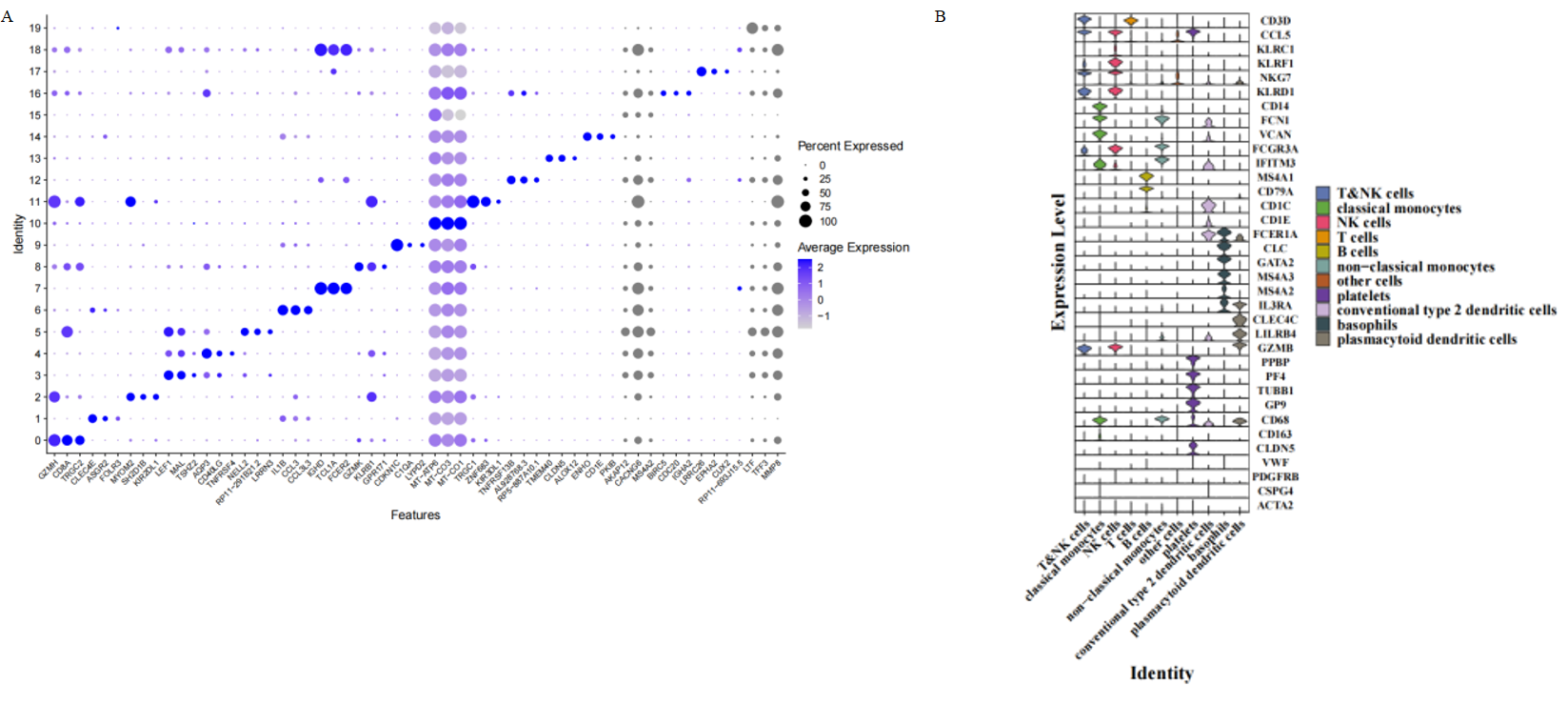

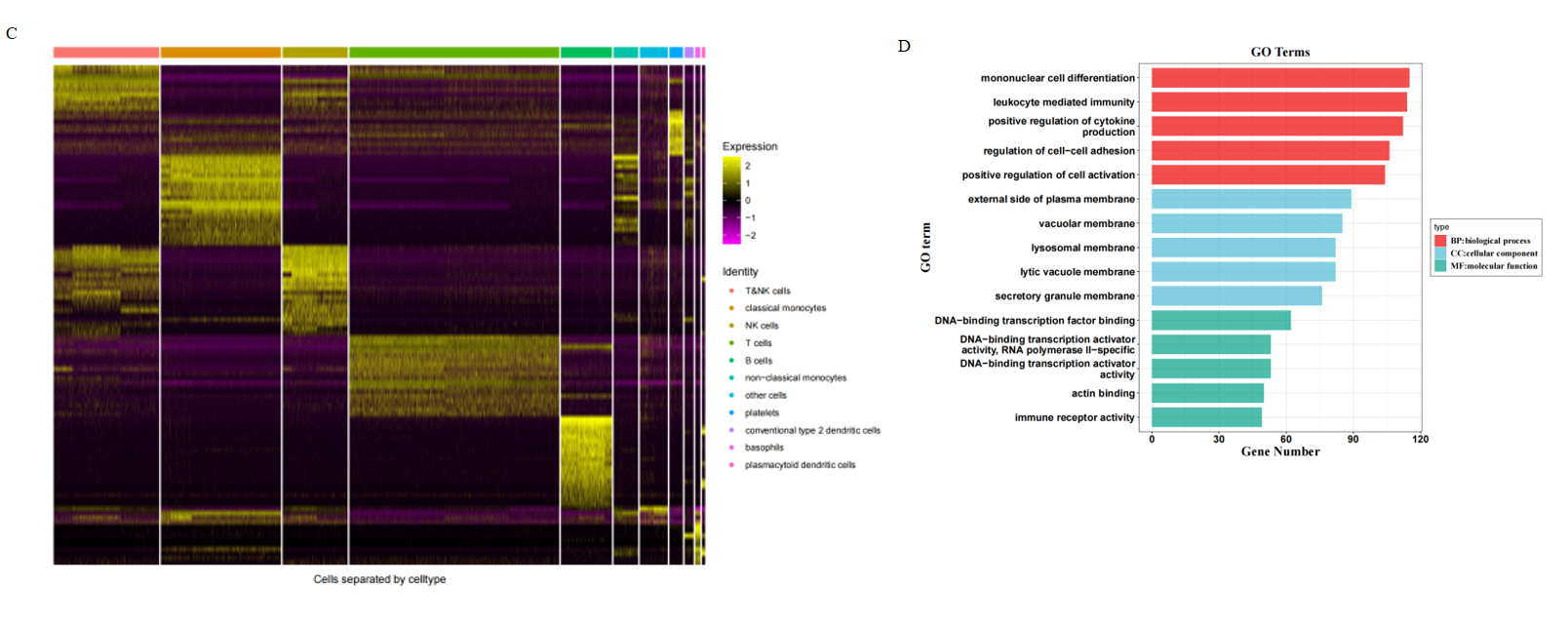
 **Figure S7.** (A) Bubble plot of marker gene expression levels; (B) violin plots of specific marker gene expression in each cell type; (C) heatmap of marker gene expression levels; (D) GO enrichment results of marker genes.
